# Structure, Dynamics, and Interaction of *Mycobacterium tuberculosis* (*Mtb*) DprE1 and DprE2 Examined by Molecular Modeling, Simulation, and Electrostatic Studies

**DOI:** 10.1371/journal.pone.0119771

**Published:** 2015-03-19

**Authors:** Isha Bhutani, Saurabh Loharch, Pawan Gupta, Rethi Madathil, Raman Parkesh

**Affiliations:** Institute of Microbial Technology, Council of Scientific and Industrial Research, Chandigarh 160036, India; Bioinformatics Institute, SINGAPORE

## Abstract

The enzymes decaprenylphosphoryl-β-D-ribose oxidase (DprE1) and decaprenylphosphoryl-β-D-ribose-2-epimerase (DprE2) catalyze epimerization of decaprenylphosporyl ribose (DPR) todecaprenylphosporyl arabinose (DPA) and are critical for the survival of *Mtb*. Crystal structures of DprE1 so far reported display significant disordered regions and no structural information is known for DprE2. We used homology modeling, protein threading, molecular docking and dynamics studies to investigate the structural and dynamic features of *Mtb* DprE1 and DprE2 and DprE1-DprE2 complex. A three-dimensional model for DprE2 was generated using the threading approach coupled with *ab initio* modeling. A 50 ns simulation of DprE1 and DprE2 revealed the overall stability of the structures. Principal Component Analysis (PCA) demonstrated the convergence of sampling in both DprE1 and DprE2. In DprE1, residues in the 269–330 area showed considerable fluctuation in agreement with the regions of disorder observed in the reported crystal structures. In DprE2, large fluctuations were detected in residues 95–113, 146–157, and 197–226. The study combined docking and MD simulation studies to map and characterize the key residues involved in DprE1-DprE2 interaction. A 60 ns MD simulation for DprE1-DprE2 complex was also performed. Analysis of data revealed that the docked complex is stabilized by H-bonding, hydrophobic and ionic interactions. The key residues of DprE1 involved in DprE1-DprE2 interactions belong to the disordered region. We also examined the docked complex of DprE1-BTZ043 to investigate the binding pocket of DprE1 and its interactions with the inhibitor BTZ043. In summary, we hypothesize that DprE1-DprE2 interaction is crucial for the synthesis of DPA and DprE1-DprE2 complex may be a new therapeutic target amenable to pharmacological validation. The findings have important implications in tuberculosis (TB) drug discovery and will facilitate drug development efforts against TB.

## Introduction

The global health crisis caused by TB is greater than ever, affecting both developing and developed countries with the disturbing rise of multidrug resistant (MDR) and totally drug resistant (XDR) *Mtb* strains[1]. TB infection is unique as *Mtb* can survive in a dormant state, commonly called ‘persister’, for a longer period of time inside the host[2,3,4].Approximately one-third of the world’s population carries this latent form of *Mtb*, as a direct consequence of persistence[5,6]. Latent *Mtb* can lead to TB if the immune system is compromised, for example, in cases of HIV or other immunosuppressive diseases[7]. Current treatment for TB involves a multi drug regime that needs to be continued for at least 6 months[8]. To combat TB, it is critical to develop drugs that work within a short time and can successfully eradicate latent bacilli. The crisis is unprecedented as only one new drug *i*.*e*. Bedaquiline has been approved for TB in the last four decades[9]. The drug is effective against *Mtb* including MDR strains but is also associated with risks of nausea, arthralgia, headache, hemoptysis, chest pain and even death [10,11,12]. There is an urgent need to explore novel drug targets and to design effective novel drugs for TB.

The dynamic cell wall of *Mtb* is a promising drug target as it shields the bacillus from stress and is critical for its virulence and pathogenicity[13]. The cell wall is highly intricate with components ranging from lipids, glycolipids and peptides, forming an impermeable barrier. Two critical cell wall constituents are the peptidoglycan-arbinogalactan-mycolic acid complex (PAM) and lipoarabinomannan (LAM). In PAM complex, the peptidoglycan is covalently attached to arabinogalactan, which in turn forms an ester linkage with the mycolic acid. The peptidoglycan layer is composed of β-(1,4)-linked alternating sugar units of N-acetylglucosamine and N-acetylmuramic acid where N-acetylmuramic residues are cross-linked *via* a tetrapeptide[14].The arabinogalactan layer consists of galactan and arabinan residues. The mycolic acid layer consists of α,β-saturated hydroxyl acids and a meromycolic acid organic moiety attached to different functional groups (ethoxy, methoxy and keto) as well as to cyclic structures such as cyclopropanes[15]. The PAM complex is highly involved in *Mtb* pathogenesis, virulence, and lethalilty. LAM is a polysaccharide composed of D-arabinofuranose (Araf) and mannopyranosyl residues.Arabinans, lipomannans, arabinogalactan (AG), and lipoarabinomann are essential building blocks of PAM and LAM, and are produced by diverse biosynthetic pathways. One of the crucial biosynthetic step is the production of Araf residues. Araf is generated from DPA by epimerization of the intermediate DPR[16,17]. It has been shown that without DPA both latent and virulent *Mtb* bacteria are unable to survive[18]. DprE1 is considered as a magic drug target for small molecule intervention in TB, as no human orthologue has been reported[19]. Phenotypic screening assays have identified a number of small molecules targeting DprE1 that demonstrate good antibacterial activity with no report of *Mtb* resistance. Benzothiazinones (especially BTZ043) is potent against DprE1 [18]. An analogue of BTZ043 *i*.*e*. PBTZ has synergistic effect when combined with TMC207 [20]. TCA1 and various 1,4-azaindoles have been shown to inhibit DprE1 through non-covalent mode of inhibition [21,22,23]. These inhibitors have confirmed DprE1 as a promising target [18,20,22,23].It has been proposed that both DprE1 (Rv3790/MT3898) and DprE2 (Rv3791) are essential for the biosynthesis of DPA from DPR[17].Recent genetic studies have demonstrated the importance and essentiality of DprE2 for*Mtb*survival[24]. Thus, both DprE1, DprE2 and DprE1-DprE2 complex are excellent drug targets and could be exploited by targeting with small molecule therapeutics. Crystal structures of DprE1 (from *Mtb* and *M*. *smegmatis)* have been reported recentlyin both apo and ligand-bound forms[20,21,25,26,27]. The reported crystal structure has two major disordered regions for which no electron density was observed. The crystal structure of DprE2 has not yet been determined.

The use of computational tools such as molecular dynamics and modeling is increasingly being used to understand the protein complexes involved in *Mtb*infection. For example, homology modeling and molecular dynamic studies have been used to predictand validate the *Mtb*-MurD structure. The docking analysis has been used to highlight the active site residues of *Mtb*-MurD [28]. Docking has been used to predict the binding interactions of ligands with*Mtb*-OMP Decase [29]. Recently, the structures of *Mtb*-SigF and *Mtb*-Usfx were evaluated by comparative modelling techniques and active sites of the two proteins were identified[30]. Molecular docking has also been used to understand the interaction mechanismbetween *Mtb* Eis and DUSP16/MKP-7. The docked model suggested that the binding of substrate depends not only on the favorable geometric arrangement but also on the electrostatic complementarity [31]. Molecular docking simulations have been used to identify the role of receptor flexibilty in case of InhA enzyme from *Mtb*. The snapshots were collected from three different MD simulations of the InhA enzyme and their docking was explored to three different inhibitors of InhA. The study suggests that various ligand conformations can be accommodated by flexible receptor model [32]. Mutational studies using MD have been performed on NADH-InhA to understand the underlying mechanism of drug resistance. The study revealed that in wild-type InhA-NADH complex, the NADH molecule remains in bound conformation whereas in mutant complexes, the conformational changes in NADH pyrophosphate moiety results in its weak interactions with the binding site [33]. These computational methods are advantageous as they enable rapid and cost-effective solutions to predict the biology of a protein or complex.

The objective of the present work is to use modeling and molecular dynamics studies to understand the disordered regions of DprE1 and elucidate a possible role and mechanism of the DprE1-DprE2 interaction involved in the catalysis of DPR to DPA. In order to understand the structural basis of the substrate recognition and to characterize the DprE1-inhibitor and DprE1-DprE2 interaction, we performed homology modeling, MD simulations, and docking studies. This will have implications for the rational design and development of novel antimicrobial agents with improved potency and activity for the effective control of TB, including persistent or latent forms.

## Methods

### Molecular modeling of DprE1

The amino acid sequence (461 residues) of the target protein, *Mtb*DprE1(UniProt code: *Mtb* P72056), was retrieved from the UniProt protein sequence database (http://www.uniprot.org). The three-dimensional model was constructed by a comparative modeling strategy using the template structures of DprE1 in complex with inhibitors CT319[25] and TCA1[21] (PDB codes: 4FDO, chain A, and 4KW5, chain A). The reported crystal structures display two large disordered regions at residues 269–297 and 316–330. To account for these structural disorders, homology modeling was performed. Sequence alignment was derived with the Modeller 9.12/SALIGN module[34] and carefully evaluated. It was then used as an input to generate viable models by the derivation of restraints from the given related structures and their alignment with the target sequence. The crude models were further refined using the DOPE (Discrete Optimized Protein Energy) assessment method implemented in Modeller 9.12 as a guide.

### Molecular modeling of DprE2

The amino acid sequence (254 residues) of *Mtb*DprE2 (UniProt code: P66783) was retrieved from the UniProt protein sequence database. To the best of our knowledge, no X-ray crystal structure has been reported for DprE2. To generate three-dimensional models, we used a protein threading alignment program I-TASSER [35]. The I-TASSER algorithm first gives a prediction of the secondary structure from the sequence. Firstly, the primary sequence and predicted secondary structure are searched against a PDB library database of solved protein structures to identify the best possible templates. I-TASSER retrieves template proteins with similar folds from the PDB library, and in cases where no similar structures are found, *ab inito* modeling is used to build a three-dimensional structure.The templates are then split into fragments and reassembled into full-length models, which are further optimized for H-bonding and steric clashes to predict full atomic models. This structure-fragment assembly approach is considered to be the best for generating high quality models for proteins for which homology modeling is not possible[36].The confidence score (C-score) was used to evaluate the quality of the generated models. C-scores fall within the range of −5 to +2 with scores above −1.78 indicating correct model topology. We used EndScript 2.0 [37,38] for secondary structural analysis.

### Structures quality check

The programs Verify-3D[39], ERRAT[40], PROCHECK[41] and ProQ[42]were used to perform quality checks on the DprE1 and DprE2 structures generated. Verify-3D was used to check the structures and sequence compatibilities.ERRAT was used to verify the overall quality whereas PROCHECK was employed for assessing the overall stereochemical quality of the generated models. The DprE1 and DprE2 structures were also analyzed using ProQ which is a neural network based predictor based on machine-learning techniques to assign different types of quality measures to the developed models. We used the SolvX server [43] to measure the solvent accessibility of all the residues of DprE1 and DprE2. The what-if “fine check quality control” analysis was used to review the compatibility of the amino acids in their local environment [44]. The initial structure as well as the refined structures generated from MD simulations of DprE1 and DprE2 were analysed by what-if. The loop residues in the disallowed regions in PROCHECK were optimized before performing simulations by using the ModLoop webserver[45]

### Secondary structure prediction

For a comprehensive secondary structure prediction, a consensus was built for the DprE2 sequence by taking into account the result of tools such as PSIPRED[46], PSSpred[47], YASPIN[48], Scratch[49], and Gor4[50]. The generated model of *Mtb*DprE2 was checked for accuracy of the secondary structure.

### Sequence-based interaction of DprE1 and DprE2

The interaction of DprE1 (Rv3790/MT3898) with DprE2 (Rv3791) was confirmed by using the STRING 9.05 (http://string-db.org), interaction database[51]. STRING employs a set of prediction algorithms to assess the interaction between the query protein and its partner based on a database of 5 million proteins from 1133 different organisms[52]. It identifies potential interacting protein partners based on existing information about other organisms, genetics, phylogenetic co-occurrence, and functional association. The output is quantified by a confidence score matrix.

### Molecular dynamics simulationsof DprE1 and DprE2

Simulations for both DprE1 and DprE2 were performed using GROMACS 4.5.5[53]with AMBER-03 force field[54] running on a Linux cluster (64 bit processor) with eight processors having ten cores each. Simulation parameters included solvation using cubic boxes filled with SPC216 water molecules, periodic boundary conditions, TIP3P water, charge-neutralizing Na^+^ and Cl^−^ ions, and PME for electrostatic interaction. Van der Waals non-bonded interaction cutoff of 12Å with a 2fs time step was used during simulations. Additionally, 0.1 M NaCl was established to mimic the physiological conditions. The minimization was performed using the steepest descent algorithm and the benchmark for convergence was either the maximum force of the system (less than 10 kJ/mol/nm) or no drastic energy changes during minimization steps. The equilibration for dynamic simulations was performed under NVT which was followed by NPT. Simulations were equilibrated in the NVT ensemble in which the system was slowly heated and the protein was subjected to positional restraint to facilitate the relaxation of solvent molecules around the protein structure. These restraints were removed and the system was coupled to a heat bath at 310 K using a Berendsen thermostat[55] and equilibrated for 100 ps. For the NPT simulation ensemble, the Berendsen was replaced by a Nose-Hoover thermostat[56], which produces a more correct ensemble of kinetic energies, and the Parrinello-Rahman[57] method was used to couple pressure isotropically to a value of 1.0 bars with a compressibility of 4.5x10^−5^ bar^−1^ for 500 ps. The system was then subjected to a 50 ns production dynamics (PD) simulation run using the same integrator, pressure, and temperature. The PD simulations were initiated by using velocities and coordinates obtained from previously run equilibration simulations at a temperature of 310 K and 1 atm pressure. Bond length constraints were based on the Linear Constraint Solver (LINCS) algorithm[58]. Three MD simulations of 50 ns each were performed for DprE2 system by varying the random seed for initial velocity generation. MD trajectory analysis was performed using Gromacs utilities and some external programs. All the graphs were plotted using Grace (http://plasma-gate.weizmann.ac.il/Grace/).

### DprE1 and DprE2 ensembles for protein-protein docking

The DprE1 and DprE2 50 ns MD trajectories were used for generating an ensemble for docking. The idea behind ensemble docking is to automatically optimize and select a conformation that best fits the ligand. Protein flexibility is demanding because of various degrees of flexibility ranging from side chain rearrangements to global motions [59,60]. Ensemble based docking introduces protein flexibility by including conformations that can span large degrees of flexibility in the protein[61,62,63]. The approach is to dock the ligand/protein into multiple protein conformations such that the best scored conformation could be found. In this way, the docked complexes could be improved iteratively by docking flexible ligands into multiple conformations. Hence it would be easier to obtain deep understanding of DprE1-DprE2 binding mechanism.

Clustering analysis was performed to generate an ensemble containing representative DprE1 and DprE2 structures throughout the simulation. It is not possible to dock all conformations of MD. A cautiously selected ensemble of protein conformations from the MD trajectorycould be more useful. For the DprE1 and DprE2 MD simulation, protein conformations were clustered with the RMSD cut-off of 1.8 Å and 2.2 Å. This was done using gromos method as implemented in the g_cluster tool of Gromacs [64]. A conformation is added to aclusterwhen its distance to any element is less than the given cutoff. Once a cluster is formed, the protein conformations of the first cluster are removed and the process is repeated for the rest. The clusters were ranked according to their size and the most populated structure was considered as the top cluster. After all the clusters are formed, the conformation with the smallest average distance to the others was considered to represent the cluster. These representative structures (the structure with smallest RMSD from all other structures) from each cluster were selected for the docking ensemble.

### DprE1-DprE2 docking and molecular dynamics of the binary complex

To analyze the DprE1-DprE2 interaction, protein-protein docking was performed using ClusPro 2.0 [65]. For docking analysis, we used the lowest energy metastable conformations of DprE1 and DprE2 as obtained from MD due to the stereochemical quality of the structures. It has been demonstrated that the lowest energy model is close to the near native structure [66,67,68]. We used this correct stereochemical structures for protein-protein docking as it has been demonstrated that lowest energy sterochemically correct structure provides comparable results as of MD ensemble[69].ClusPro generate putative complexes taking into account the electrostatic, hydrophobic, and van der Waals interactions. The interactions were ranked according to their clustering properties. The ClusPro docking server employs PIPER’S efficient FFT (Fast Fourier Transformation) docking approach [65]. The FFT docking algorithm is further optimized with accurate pairwise potentials to significantly reduce the number of initial poses, thus eliminating the need for additional rigid-body filters and computationally demanding electrostatic calculations.The docked complex obtained from ClusPro was verified with GRAMM-X [70] and PatchDock [71]. GRAMM-X works upon Fast Fourier Transformation methodology to generate structures and further refines them from atomic clashes and knowledge based scoring. PatchDock was used to dock models based on the shape complementarity of soft molecular structures. The docked candidates from PatchDock were then used by FireDock to optimize and rescore the top 10 candidates by restricting side chain flexibility of the interacting surface [72]. To refine the resultant complex structure obtained from docking, simulations of the binary complex were performed for 60 ns with the same operational parameters as discussed in the DprE1-DprE2 simulation methodology. The docking performed using the metastable lowest energy structures of DprE1 and DprE2 was considered further for MD simulations.

To incorporate flexibility into docking and to reconfirm the results obtained from the lowest energy structures docking, ensemble based docking was performed. The 50 ns MD ensemble was used for DprE1 and DprE2 docking. The representative structures (structures with smallest RMSD from others in the cluster) from all seven clusters of DprE1 and DprE2 were included in docking. The docking is performed from various combinations of starting conformations of DprE1 and DprE2. Each protein conformation of DprE2 was docked into all representative conformations of DprE1 using ClusPro.The representative structures from all the clusters for both DprE1 and DprE2 were included in docking. We selected a highly populated cluster and isolated the lowest energy structure for the analysis. The top poses were selected on the basis of energy and by visualization. The protein-protein interactions in the binary complex were examined using PyMol and Protein Interaction Calculator (PIC) web server (http://pic.mbu.iisc.ernet.in) [73].

#### DprE1-DprE2 contact map

To reconfirm the binding site interface of DprE1 and DprE2, intermolecular contact map was made using COCOMAPS [74]. It has been shown that intermolecular map has the ability to recognize the interaction surface properly[75]. We used the tool “COCOMAPS” to analyze the contacts at the interface of DprE1 and DprE2. The DprE1-DprE2 lowest conformation obtained after MD was used as an input.

### DprE1-inhibitor interactions

Various BTZ inhibitors such as BTZ043, BTZ046 *etc*. are known to target *Mtb* DprE1 [18]. BTZ043 is the most potent of these inhibitors. A crystal structure of *M*. *smegmatis* DprE1 in complex with BTZ046 (hydroxylamine derivative of BTZ043) is reported [26]. However, there is no crystal structure available for *Mtb* DprE1 in complex with BTZ043. In this context, it would be useful to dock BTZ043 into DprE1. Ensemble based docking was performed to find an optimal DprE1 confirmation to fit BTZ043.

#### Receptor and ligand preparation

The representative structures (structure with smallest RMSD from all other structures of the cluster) selectedfrom the DprE1 ensemble were used for docking. The DprE1 ensemble for DprE1-BTZ043 interaction studies was formed as described in “DprE1 and DprE2 ensembles for protein-protein docking” section. The coordinates of FAD were taken using the available crystal structure of *M*.*smegmatis* DprE1 in complex with FAD and BTZ046 (PDB ID: 4F4Q). To prepare the receptor for docking, water molecules were removed, polar hydrogen atoms were added, Kollman’s charges were assigned and the non-polar hydrogens were merged. [26]. The representative conformation of each cluster was prepared using AutoDock tools [76]. The ligand BTZ043 was downloaded from ChemSpider (http://www.chemspider.com/). The antechamber program [77], accessible through the Chimera suite (http://www.cgl.ucsf.edu/chimera), was used to derive point charges and the BTZ043 structure was minimized by steepest descent followed by conjugate gradient protocol to remove steric clashes.

#### Docking

The binding pose of BTZ043 into *Mtb* DprE1 was predicted by AutoDock 4.2, a widely used software for molecular docking [76]. The binding site cavity for *Mtb* DprE1 was defined by His-132, Gly-133, Lys-134, Leu-317, Val-365, Lys-367, Phe-369, Cys-387 and Lys-418 based on the active site residues of the templates (PDB ID: 4F4Q, 4FDO, 4FDN) [25,26]. In docking simulation, we used semi flexible docking protocols in which the defined binding site residues of DprE1 were treated flexible whereas the others were kept rigid. The ligand BTZ043 was also kept flexible to explore an arbitrary number of torsional degrees of freedom spanned by the translational and rotational parameters. AutoDock calculates the binding energies between protein and ligand using atom affinity potentials which are pre-calculated on grid maps using AutoGrid. A grid box of dimensions 58x60x56 in the X, Y, and Z directions, with a spacing of 0.375 Å, was used to cover the active site of *Mtb* DprE1 in all the conformations. BTZ043 was docked using the Lamarckian Genetic Algorithm (LGA) in the conformational space defined by the grid. The LGA parameters were of population size: 150, mutation rate: 0.02 and crossover: 0.8. The maximum number of energy evaluations was set to 2.5 million and maximum number of generations was kept 27000. The BTZ043 conformations were ranked in the order of increasing docking energies and clustered with a RMSD cutoff of 2 Å. The top poses were selected from each docking simulation. Further, the best pose was selected based on binding energy and clustering. This conformation was regarded as the binding conformation between BTZ043 and DprE1. Interactions were analyzed using AutoDock Tools [76] and Ligplot+ [78].

### Principal component analysis (PCA)

PCA systematically reduces the dimensionality of a complex system and can characterize the cumulative and overall motion of the protein system. The first principal component typically encompasses the largest root mean-square fluctuation (RMSF) [79,80,81]. Using this approach, we have focused on revealing atomic fluctuations in DprE1 and DprE2. The fluctuations in the system are the result of the correlations between the motion of particles, which is directly related to the behavior or function of the protein.

Eq. 1 refers to the covariance matrix.

$$C_{ij}=\langle \left(x_{i}-\langle x_{i}\rangle \right)\left(x_{j}-\langle x_{j}\rangle \right)\rangle$$ (1)

In this equation x_i_ and x_j_ represent mass-adjusted Cartesian coordinates of particles i and j, respectively, whereas 〈〉 is the ensemble average of all MD simulation structure samples over the course of the simulation. Our analysis was restricted to Cα atoms, as it is less perturbed by statistical noise and provides significant characterization of the essential space motions [82]. Eq. 2 diagonalizes the symmetric matrix C essential in solving the eigenvalue problem, where A represents the eigenvectors and λ is the associated eigenvalue.

$$A^{T}CA=\lambda$$ (2)

The associated eigenvalue gives the sum of the fluctuations described by the collective motion per atom and, thus, is a measure of the total motility associated with an eigenvector. The PCA calculations were analyzed using g_covar and g_anaeig utilities of GROMACS. To estimate the essential subspace sampled by a protein system during the simulation run time, eigenvector motion is visualized by projecting each frame of the molecular dynamics simulation trajectory onto the eigenvector. The essential degrees of freedom were extracted from the equilibrated portions of the trajectories. Highest ten eigenvalues were obtained by the diagonalization of the covariance matrix of the Cα atom variance. For bound states of DprE1 and DprE2, the proteins were extracted from the complex MD trajectory.

#### Convergence of eigenvectors

To investigate the convergence of the essential subspaces in time and to evaluate their similarity, the whole trajectory was split into two halves of 25 ns each and the root mean square of inner products (RMSIP) was computed. Eq. 3 represents RMSIP, where n_i_ and n_j_ are the i^th^ and j^th^ eigenvectors of two sets, respectively [83].

$$RMSIP={\left[\frac{1}{10}\sum_{i=1}^{10}\sum_{j=1}^{10}{\left(n_{i}n_{j}\right)}^{2}\right]}^{\frac{1}{2}}$$ (3)

#### Projection of conformations onto the subspace spanned by the PCs

The eigenvectors corresponding to these eigenvalues were used to describe the essential subspace of DprE1, DprE2 and DprE1-DprE2 complex throughout the 50 ns MD simulation.The eigenvalues represent the amplitude of eigenvector along the space whereas the concerted motion of protein is the result of atom displacement along the eigenvector. We compared the mobility of unbound DprE1 and DprE2 projected on DprE1 and DprE2 extracted from the DprE1-DprE2 50 ns complex trajectory respectively.

#### Cross-correlation plots

The cross-correlation matrices were created by ProDy [84]to represent the dynamic cross-correlated displacements of Cα atoms of DprE1 and DprE2 in the bound and unbound MD trajectories. We took 2350 snapshots extracted from the 3ns to 50 ns trajectory. The foremost step was the superimposition of all frames on the initial frame of each trajectory run. The cross-correlation matrix elements for i and j Cα atoms, Cij, are defined by the equation:

$$C_{ij}=\langle \Delta r_{i}\Delta r_{j}\rangle /{\left(\langle \Delta r_{i}{}^{2}\rangle \langle \Delta r_{j}{}^{2}\rangle \right)}^{1/2}$$ (4)

The vector Δr_i_ denotes the displacement from the mean position of the i^th^ atom considering all conformations of the MD trajectory.whereas 〈〉 denotes the time average over the entire trajectory. C_ij_varies from −1 to+1 where “−1”and “+1” represent completely anti-correlated and correlated motion respectively.

#### Porcupine plot

To visualize the direction and extent of the principle motions of DprE1 and DprE2 in their bound and unbound state, porcupine plot analysis was performed. We used 50 ns trajectory for DprE1, DprE2 and DprE1-DprE2 complex. The Cα atomic motion of the topmost eigenvector of DprE1 and DprE2 was visualized using porcupine plots. The Cα atoms have arrows pointing towards the direction of motion where the cone reflects the direction and the length represents the extent of motion. The porcupine plots were generated using the tool “porcupine” and graphical images were presented using VMD.

### Electrostatic calculations

Electrostatic calculations were performed using the Poisson-Boltzmann (PB) equation as implemented in the APBS Program[85]. For preparing the system, the PDBcoordinate files of DprE1, DprE2, and the DprE1-DprE2 complex were used to assign the correct protonation state of the residues using the program PROPKA. The PQRfiles were generated using the program PDB2PQR by assigning the proper atomic radii and charged using AMBER force field[86,87].In electrostatic calculations, the lowest energy structures of DprE1, DprE2 and DprE1-DprE2 were used. The electrostatic calculations were then performed with the parameters: 1)ionic strength, 50 mM; 2) ionic radius, 1.5 Å; 3) dielectric constants (inside protein), 4.0; 4) dielectric constant in water-, 80.0. For the dielectric boundary calculations, van der Waals surface was applied.

## Results and Discussion

We have investigated molecular modeling, MD simulations, docking and PCA studies on DprE1, DprE2 and DprE1-DprE2 complex for a detailed understanding of DPA biosynthesis. The crystal structures of DprE1 and the disordered regions reported in its crystal structures were explored in depth[21,25,26,27]. In case of DprE2, three-dimensional models were generated by a protein threading approach. The three-dimensional model was subjected to MD simulations to ascertain structural information. Finally, docking and simulation studies on the two proteins were done to investigate DprE1-DprE2 complexation and the important structural features involved in DprE1-DprE2 interaction. We have also analyzed DprE1-inhbitor interactions.

### Molecular modeling of DprE1

Several crystal structures of DprE1 from *M*. *tuberculosis* and *M*. *smegmatis* have been solved[21,25,26,27].*Mtb* DprE1, consisting of 461 residues, shows two major domains: 1) a FAD binding domain (involving residues 7–196 and 413–461) and 2) a substrate-binding domain (involving residues 197–412)[25]. The FAD binding domain consists of an α/β fold, β-sheets, and several α-helices. The substrate binding domain is comprised of antiparallel β-sheets along with three α-helices that extend to the back of the enzyme. Two noticeably disordered regions (residues 269–297 and 316–330) are located in the substrate binding domain and might be involved in substrate recognition and complex formation with DprE2.

To explore the structural features of disordered regions, the homology model of DprE1was generated. The modeling involves the alignment of the target DprE1 sequence with the known three-dimensional structural templates. Modeller uses a database of many known 3D protein structures for this template modeling. The three-dimensional model of the target DprE1 sequence containing all the main and side chain non-hydrogen atoms were generated. For DprE1 comparative modeling, five models were created, and the model with the highest DOPE score was selected for MD simulations. The Ramachandran plot showed that the overall stereochemical quality of the generated model is good[41]. The Ramachandran plot for model shows that almost all the residues are in the most favored, additionally allowed, or generously allowed regions except Asp-277, which falls in the disallowed region. The three-dimensional model was further optimized using ModLoop server, which involved optimization of the loop containing Asp-277 by satisfying the spatial restraints. The Ramachandran plot of the homology model of *Mtb* DprE1 optimized by ModLoop shows that residues are now in the most favored or additionally allowed regions (S1 Fig.). There are no residues in the disallowed region, which indicates a high quality model. Verify-3D [39] analysis of the *Mtb* DprE1 model shows that the residues are located in their most favorable three-dimensional structured environment. The what-if quality report for DprE1 model before and after the MD simulation also indicates high grade model (S1A Table).The merit of the model was also confirmed by various other tools as shown in S2 Table.

The homology models of DprE1 shows that the disordered regions observed in the crystal structure(269–297, 316–330) have some structural features.The regions 292–300 and 316–327 form helical structures. During MD simulation the structural integrity of these helical regions was maintained. The region 269–286 remained disordered in the homology model, MD ensemble, and lowest energy MD structure. Root mean square fluctutations analysisfrom MD clearly showed that highest atomic flucutations are observed in this particular region further confirming this as a extremely perturbed region. It is possible that this disordered loop (269–286) will assume ordered structure upon complex formation, membrane interaction and stabilization of substrate binding.We envisage that this loop might be stabilized by small molecule inhibitor thus, this region will be a potential target for developing new inhibitors. Our rigid body and ensemble-based docking of DpE1-DprE2 indicates a major role of these loop residues in complex formation.

### DprE2 fold recognition and validation

For *Mtb* DprE2, no significant template with good query coverage and sequence identity (>30%) was found in BLAST search. We obtained five models from threading/fold recognitionand we chose the best model with the correct topology. The highest C-score for the *Mtb* DprE2 model was 0.12, which suggested correct topology. The Ramachandran plot of the model shows that most of the residues are in the allowed region, although residues Ala-70, Asn-108, Asp-209, Lys-223 and Arg-246 are found in the disallowed region. This three-dimensional model was further optimized using ModLoop server, which involved optimization of the loop containing these five residues. The Ramachandran plot of the optimized model shows that most of the residues are in the most favored or additionally allowed regions, whereas only five residues are in the generously allowed region (S2 Fig.). No residue was found in the disallowed region, which indicates a fine model. Verify-3D analysis of the *Mtb* DprE2 model shows that the residues are located in their most favorable three-dimensional structured environment. The what-if quality report for DprE2 model before and after the MD simulation is represented in the S1B Table. The report reassures good quality model before and after the refinement using molecular simulations.The accuracy of the model was cross-validated with the secondary structure consensus. The consensus was built manually to predict the secondary structure of the model as described in methods. We found that the secondary structure of the generated model was in agreement with the consensus (S3 Fig.). The merit of the generated model was further confirmed by various other tools as shown in S2 Table.

According to the sequence analysis it has been suggested that DprE2 (Rv3791) belongs to the classical short-chain dehydrogenase/reductase (SDR) family [16,87]. The SDRs are enzymes of ∼250 aa in length and are involved in the catalysis of NAD(P)(H) dependent oxidation/reductions [88]. The N-terminal region binds to NADP(H) and the C-terminal is involved in the substrate binding. DprE2 shows high sequence identity with SDRs such as *Mycobacterium leprae* (89.0% identity in 254 aa overlap) and short-chain alcohol dehydrogenase from *Mycobacterium smegmatis* (85.05% identity in 254 aa) and *Streptomyces coelicolor* (45.3% identity in 254 aa overlap) [16].

**Fig 1:**
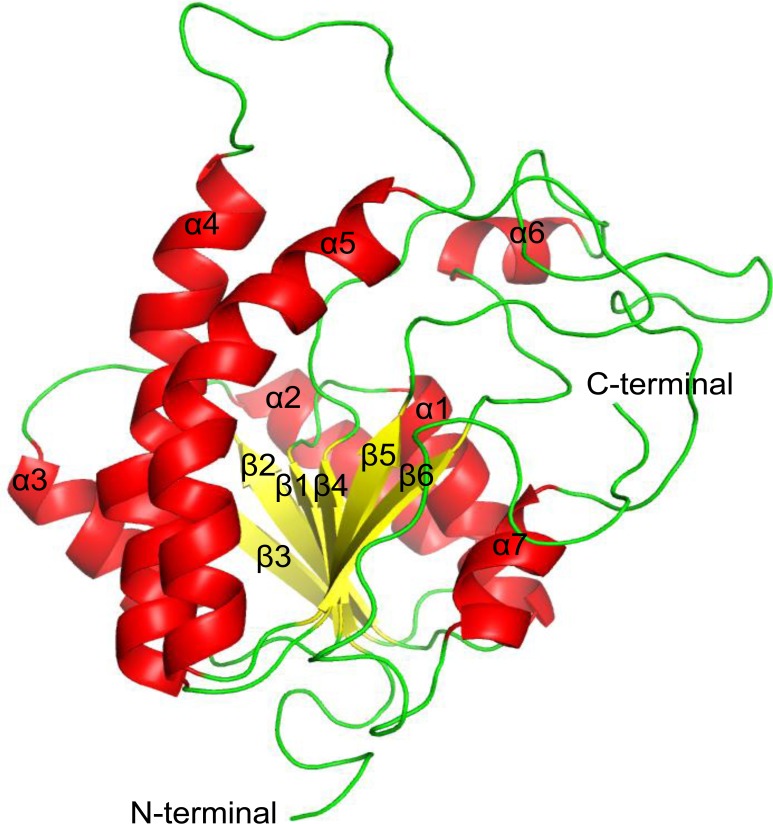
Three-dimensional structure of the*Mtb* DprE2 model. The different structural elements are shown in different colors: helices (red), β-sheets (yellow) and coils (green).

**Fig 2:**
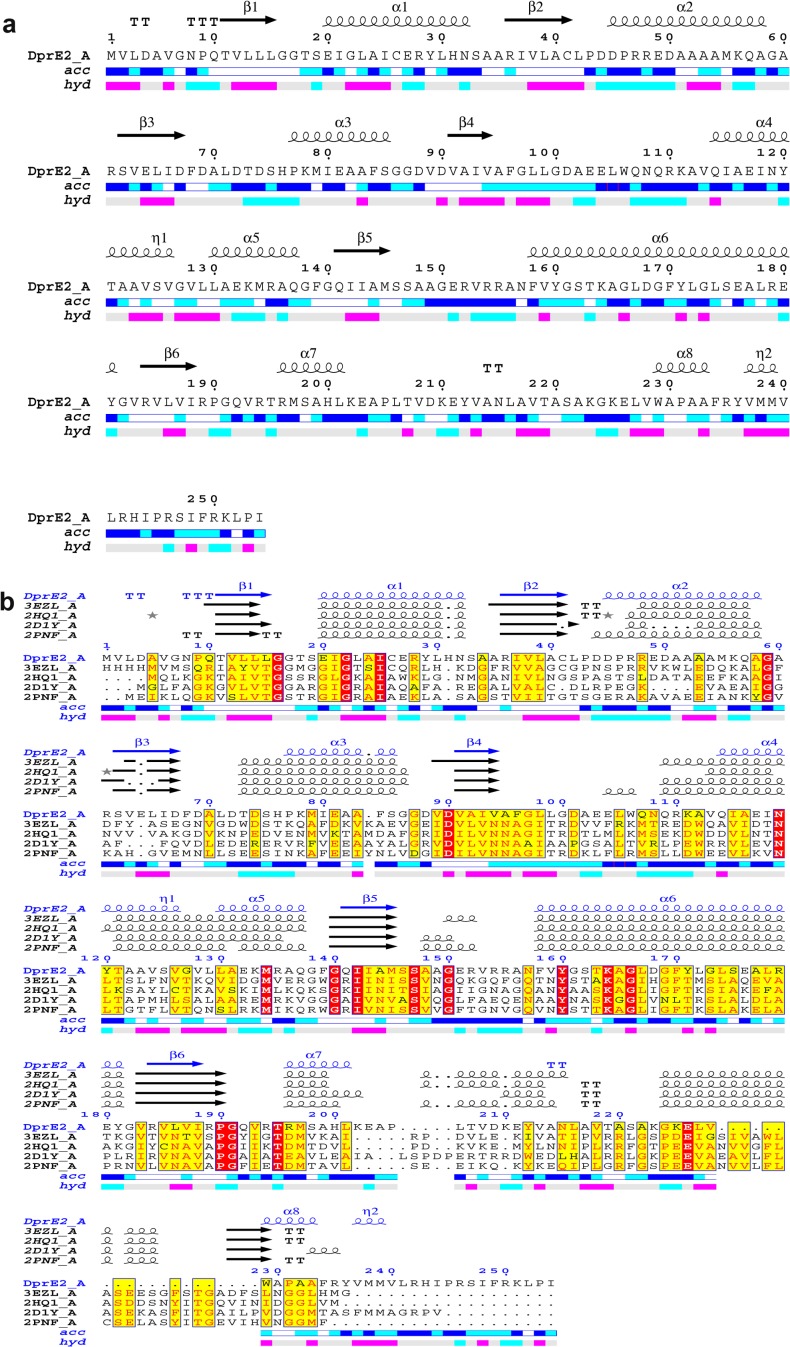
Secondary structure analysis and multiple sequence alignment between DprE2 and other members of the SDR protein family. (a) Secondary structure analysis. The α helices, β sheets and turns are represented with squiggles, arrows and TT letters, respectively.The DprE2 accessibility is shown by color codes: blue for accessible, cyan is intermediate, white is buried. The hydropathy bar is shown by a bar where pink is hydrophobic and cyan is hydrophilic. (b) The multiple sequence alignment of DprE2 with its homologous SDR proteins is shown where identical and similar residues are boxed in red and yellow, respectively.

The threaded model of DprE2 consists of eight α-helices and six β-sheets. During MD simulation one of the helix (213–218 aa) was transformed into a random coil and the core structure (lowest energy MD structure) of DprE2 adopts a typical Rossmann fold with six-stranded β-sheets (β1- β6) and seven α-helices (α1- α7) (Fig. 1). The β1- β6 include the regions 11–15, 36–41, 62–67, 91–94, 141–145 and 184–188 and are aligned parallel to each other. The α1- α7 consists of regions 20–32, 45–58, 76–85, 114–137, 158–181, 196–201 and 227–234. The ENDSCRIPT was employed for secondary structure analysis and multiple sequence alignment of DprE2 with other members of the SDR family (Fig. 2). The DprE2 model showed similar secondary structural elements in comparison to other SDR members. It was observed from predicted secondary structure analysis that helix α4 (114–137 aa) in the generated DprE2 model is split into two helixes (114–126 aa and 130–137 aa; Fig. 2A). We also observed a triad of three conserved catalytic residues namely Ser-147, Tyr-160 and Lys-164, a unique feature of SDR. Another interesting observation was high structural similarity of generated three-dimensional structure of DprE2 with SDR family members such as acetoacetyl-coA reductase (PDBID: 3EZL, rmsd: 3.43, 256 Cα) and ribitol dehydrogenase (PDBID: 2HQ1, rmsd: 3.139, 247 Cα) despite low sequence identity (Fig. 2B). This indicates that in case of SDR protein familiy, sequence diverges to a greater extent than does the structure. The three major disordered regions are observed from 95–113, 146–157 and 202–226. The secondary structure consensus analysis indicates disordered regions are 95–103, 147–157, 189–209 and 222–226. This clearly indicates that the predicted secondary structure consensus is in agreement with the generated model of DprE2.

### MD simulation shows residue fluctuations at disordered regions in DprE1 and DprE2

**Fig 3:**
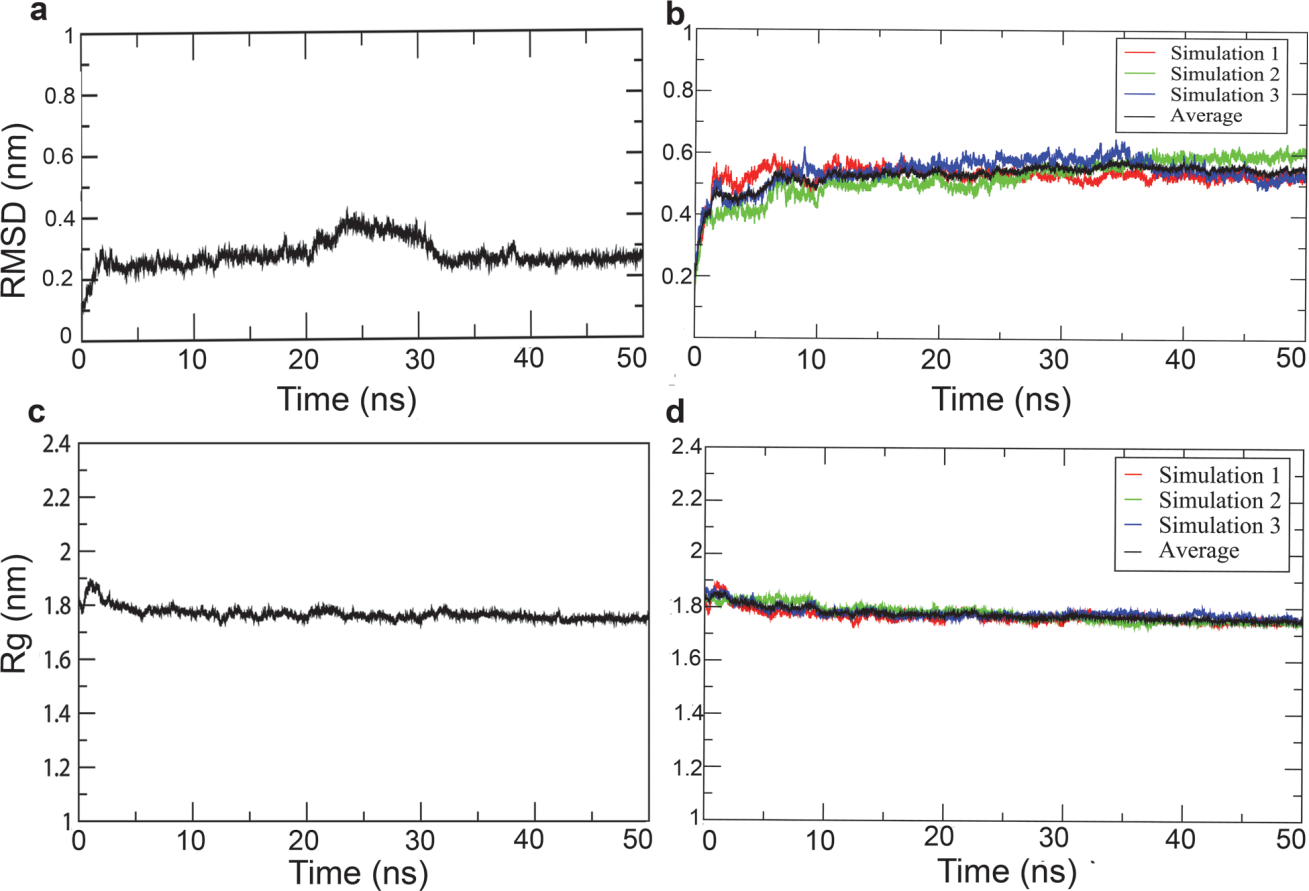
RMSD and radius of gyration (Rg) for DprE1 and DprE2 as a function of time. (a) RMSD evolution of DprE1 (b) RMSD evolution of DprE2 in triplicate (c) Rg evolution of DprE1 and (d) Rg evolution of DprE2.in triplicate. In case of DprE2 red, green and blue represent the three simulations of 50 ns each.The black line represents the average of all three simulations.

**Fig 4:**
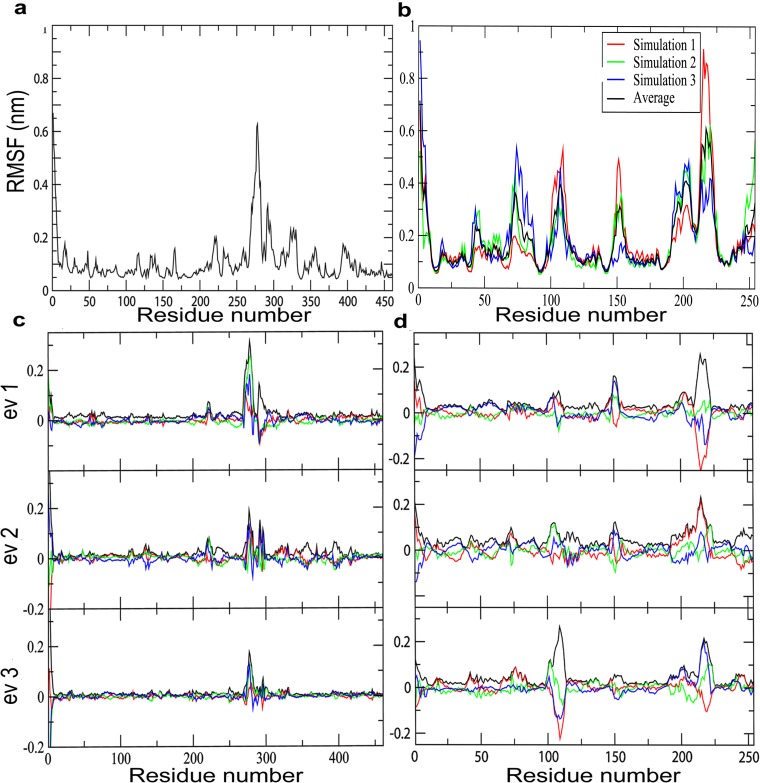
RMSF of the backbone Cα atoms versus time at 310 K for (a) DprE1 and (b) DprE2 triplicate simulation where the black line denotes the average of the three simulations. In this graph, it is observed that each simulation shows similar RMSF pattern. Projection of the Cα atom trajectory along the first three eigenvectors for (c) DprE1 and (d) DprE2 giving a more clear view of the level of residue fluctuations. The graphs depict conformationally flexible regions 269–330 in DprE1 and 95–113, 146–157, and 197–226 in DprE2. The figure depicts the first three eigenvectors to capture the dominant fluctuations in the trajectory.

The generated models of DprE1 and DprE2 were subjected to a 50 ns MD simulation protocol described in method section. The simulations resulted in relieving steric clashes, optimization of the H-bonding network and molecular geometry of the models.The simulation was run in triplicate to statistically validate the DprE2 model. The RMSD plot (Fig. 3A and 3B) shows that a stable equilibrated state is reached after 3 ns of simulation for both DprE1 and DprE2. The RMSD comparison between the initial and final structures was low, indicating relatively small movement from the initial position. The RMSD plots of the three DprE2 simulations at various points maintain similar pattern (see Fig. 3B.). The compactness of the protein structures was measured using the radius of gyration (Rg), which maintained a steady value with time (Fig. 3C and 3D). The root-mean-square fluctuation (RMSF) of Cα atoms from their time-averaged positions were analyzed for both DprE1 and DprE2 (Fig. 4A and 4B). During all three simulations of DprE2, the protein was found to be stable and showed similar patterns, validating the model statistically. The regions 269–330 in DprE1 and 95–113, 146–157 and 197–226 in DprE2 are conformationally flexible as compared to remaining regions in the trajectory.We observed highest atomic fluctuations in region 269–286 for DprE1 correlating with its disorderness.For DprE2, regions showing higher atomic fluctuations correlate with the disordered regions as observed in the 3D structure and sequence-based prediction analysis. It might be possible that these regions will be involved in complex formation with other proteins, membrane binding, substrate/inhibitors binding and substrate channeling which is important for conversion of DPR to DPA.

### Diverse sampling observed in DprE1and DprE2

**Fig 5:**
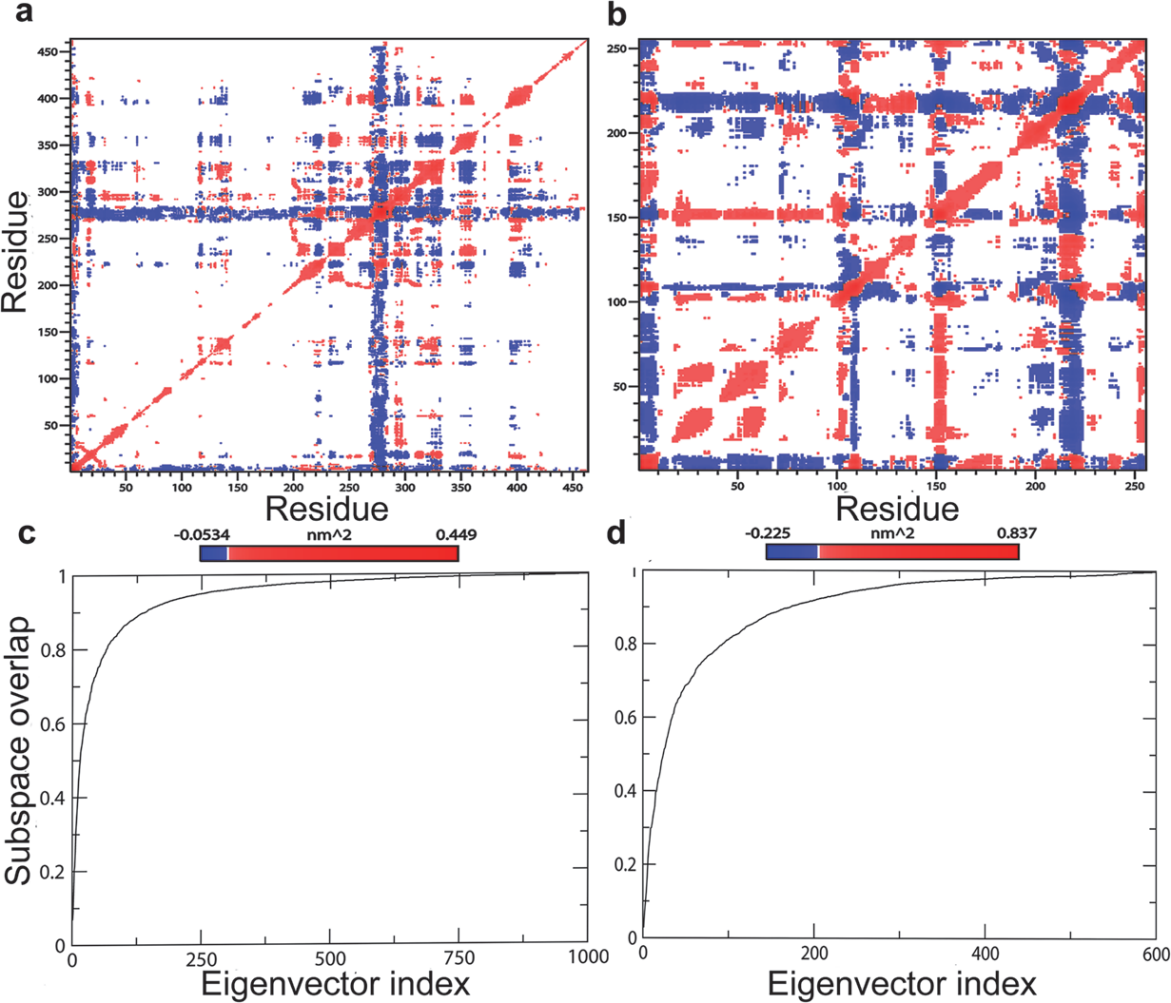
PrincipalComponent analysis of DprE1 and DprE2. Covariance matrix of the fluctuations of the Cα atoms of (a) DprE1 and (b) DprE2 during the simulationswith positive (red) and negative (blue) motions. (c) and (d) represent subspace overlap for the first 10 eigenvectors of the first half (0–25 ns) with all the eigenvectors of the second half (25–50 ns) of the simulation for DprE1 and DprE2 respectively.

PCA analysis was conducted on 50 ns trajectories for both DprE1 and DprE2 as described in method section. It has been found that the majority of protein dynamics can be successfully described by the first few eigenvectors or principal components of the entire system. To get a view of the DprE1 and DprE2 correlated residue motions, the covariance matrix of fluctuations were plotted (Fig. 5A and 5B). As shown in the figure, positive regions (light red) indicate a correlated residue movement, whereas negative regions (blue and dark blue) are generally associated with anti-correlated motions of the residues. To analyze the extent of sampling achieved within the simulation timescale (50 ns), the RMSIP[83] of two halves of the trajectories for both DprE1 and DprE2 were calculated. Eigenvectors required for RMSIP calculations were obtained from the covariance matrix. The high RMSIP values (DprE1 = 0.62; DprE2 = 0.55) indicate convergence of the conformational subspace.Previous studies have reported that RMSIP values between 0.5 and 0.7 shows adequate convergence[88,89,90]. Fig. 5C and 5D shows the subspace overlap of the first 10 eigenvectors of the first half (0–25 ns) trajectory with all the eigenvectors of the second half (25–50 ns) for both DprE1 and DprE2. This confirms that the first 10 eigenvectors of the first half of the trajectory is adequate to provide all the necessary information about the system [81,83]. Fig. 4C and 4D depicts the motion along the first three eigenvectors of DprE1 and DprE2 respectively. From Fig. 4C, it is clear in the first eigenvector plotthat the region consisting of residues 269–300 in DprE1 exhibits most dominant fluctuation. The result is further supported by second and third eigen vectors that shows identical features, implying major fluctuations in 269–300 region in DprE1. Similarly in the case of DprE2, the most dominant fluctuations are in the regions 95–113, 146–157, and 202–226 for all the three eigenvectors, suggesting DprE2 has three dominant fluctuating regions. The results indicatediverse conformational sampling in both DprE1 and DprE2.

### DprE1-inhibitor interaction analysis

Benzothiozinones (BTZ) inhibit the formation of DPA, a precursor essential for the synthesis of the arabinan chains of cell wall, leading to cell death. BTZ043 has been identified as the most potent inhibitor against *Mtb* DprE1[18]. Docking was performed to describe the binding mode of BTZ043 in DprE1.The MD ensemble based docking was used to determine the interactions.The clustering analysis generated a reduced set of conformations from the DprE1 MD trajectory.A total of seven clusters were obtained, the first being the most populated whereas the seventh contained only three conformations. The representative structures from the top six clusters were included in the docking. The coordinates of FAD were taken from the crystal structure of DprE1-BTZ046 containing FAD [26]. This DprE1-FAD complex was used for docking with BTZ043.

**Fig 6:**
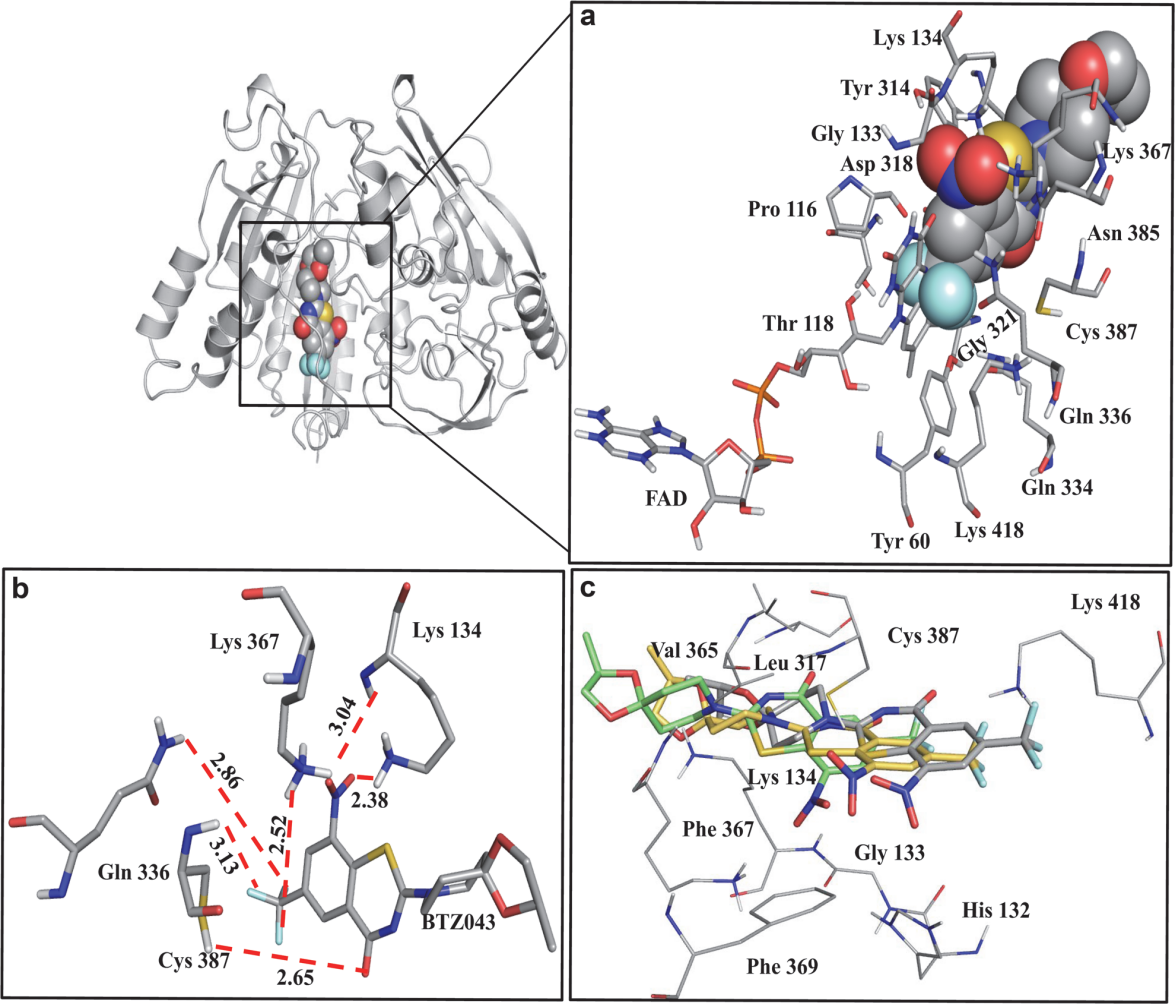
Ensemble based docking of BTZ043-DprE1 complex. (a) Cartoon representation of the DprE1 binding site occupied by BTZ043 (left) and BTZ043 interactions within the binding site (inset). (b) H-bond interaction of BTZ043 with DprE1 residues is shown by red dashed lines. (c) Superimposition of the top three poses occupied by BTZ043 into the active site of DprE1.

The best docking pose was selected based on the binding energy and by visual inspection of the binding pocket. The binding energies (predicted by AutoDock) of all six DprE1-BTZ043 complexes, are shown in S3 Table. The top pose represents the correct binding mode of BTZ043 to DprE1 with a binding energy of −11.49 kcal/mol. Fig. 6A represents the docked pose demonstrating the binding positionof DprE1 with BTZ043. Fig. 6B illustrates residues involved in hydrogen bonding. The top three ligand poses in the active site of DprE1 are shown in Fig. 6C. The figure shows that all the ligand poses bind in the same cavity. The data suggests stabilization of BTZ043 at the binding pocket by H-bonding, hydrophobic and ionic interactions. Two hydrogen bonds of length 2.38 Å and 3.04 Å were observed between oxygen atoms of NO_2_ group in BTZ043 and hydrogen atoms of the residue Lys-134. The three flourine atoms of BTZ043 formed hydrogen bonds of length 2.52 Å, 2.86 Å and 3.13 Å with hydrogen atoms of the residues Lys-367, Gln-336 and Cys-387 respectively. Additionally, a hydrogen bond of length 2.65 Å was observed between oxygen atom of benzothiazinone ring and hydrogen atom of Cys-387. BTZ043 also established a network of hydrophobic interactions with amino acid residues like Tyr-60, Gly-133, Pro-116, Thr-118, Tyr-314, Asp-318, Gly-321, Gln-334, Asn-385 and Lys-418. BTZ043shows additional interaction with the flavin moiety of FAD molecule.We also analyzed and compared our docking results with other known DprE1 inhibitor interactions. S4 Table. shows the comparison of *Mtb* DprE1-BTZ043 interactions with other known inhibitors BTZ046, CT325, CT319, TCA1 and PBTZ169 [20,21,25,26]. The table shows the unique and common interactions of reported DprE1-inhibitor complexes compared to *Mtb* DprE1-BTZ043. On comparing our results to the binding interactions of DprE1 with reported inhibitors, we observed that the binding site residues Tyr-60, Gly-133, Lys-134, Tyr-314, Gln-334, Gln-336, Lys-367, Asn-385, Cys-387and Lys-418 in DprE1 *Mtb* weresimilar. However, we found four novel residues: Pro-116, Thr-118, Asp-318 and Gly-321 participating in the *Mtb* DprE1-BTZ043 binding. Cys-394 and Lys-425 in *M*.*smegmatis*has been demonstrated as important catalytic residuesfor the binding of the BTZs [26]. Cys-387 and Lys-418 in *Mtb* in DprE1 correspond to Cys-394 and Lys-425 in *M*.*smegmatis* respectively. Our results also indicate involvement of Cys-387 and Lys-418 in the binding of BTZ043.The DprE1-BTZ043 model of *Mtb* provides information for developing and accessing the structural studies of *Mtb* DprE1 aimed at elucidating our understanding of the protein inhibitor binding.

### Do DprE1-DprE2 interact?

Recently, it has been suggested that DprE1 interacts with DprE2 during the biosynthesis of DPA, but no theoretical or experimental evidence has been shown till date[17,24]. As preliminary step, we performed sequence based interaction analysis using STRING 9.05[51] to find potential interacting partners of DprE1. The result showed that DprE2 is one of the interacting partner with confidence score of 0.999.It suggests a strong interaction between DprE1 and DprE2 and the complex formation might be important for the biosynthesis of DPA.

#### Rigid body docking of DprE1-DprE2 complex

Toexplore the specific DprE1-DprE2 interactions, docking was perfrormed.We used the balance scoring function (ClusPro) to perform docking of the DprE1 and DprE2 proteins, as this is considered to be better if there is no information about the interface residues. The ClusPro docking was also verified by GRAMM-X [70] and PatchDock [71] (S4 Fig.). To analyze the docked complex, we used Protein Interaction Server (PIC) for analysizng interactions at the protein-protein interface. PIC studies reveals that the interface is stabilized with hydrogen bonding, ionic and hydrophobic interactions (S5 and S6 Tables). Residues Asp-277 and Lys-299 of DprE1 are involved in ionic interactions with Arg-47 and Glu-104 of DprE2 respectively. Main hydrogen bonding partners, DprE1 to DprE2 are Asp-277 to Arg-47, Leu-272 to His-200, Val-278 to Arg-196, Pro-280 to Arg-196, Asn-281 to Leu-99, Gly-282 to Asp-101, Lys-299 to Asn-108, Ala-326 to Asp-72, Ala-326 to Thr-73 and Tyr-327 to Thr-73. Similarly, the hydrophobic interaction partners, DprE1 to DprE2 are Leu-273 to Met-197, Pro-276 to Leu-98, Pro-280 to Leu-98, Leu-283 to Ala-102, Ile-292 to Ala-112, Phe-320 to Ile-118, Trp-323 to Phe-96, Trp-323 to Ala-122, Phe-279 to Leu-99, Pro-280 to Leu-99, Leu-283 to Met-197, Tyr-287 to Leu-98, Tyr-287 to Ile-115, Tyr-287 to Val-159, Ile-292 to Trp-106, Leu-295 to Trp-106, Trp-296 to Trp-106, Trp-323 to Ile-118, Tyr-327 to Ile-118 and Tyr-327 to Ala-122. The ionic interaction partners, DprE1 to DprE2 are Asp-277 to Arg-47, Glu-322 to Arg-47 and Lys-299 to Glu-104.The data implies that the key residues participatingin DprE1-DprE2 complexation are from the disordered regions of DprE1.

#### Molecular dynamics of DprE1-DprE2 complex

**Fig 7:**
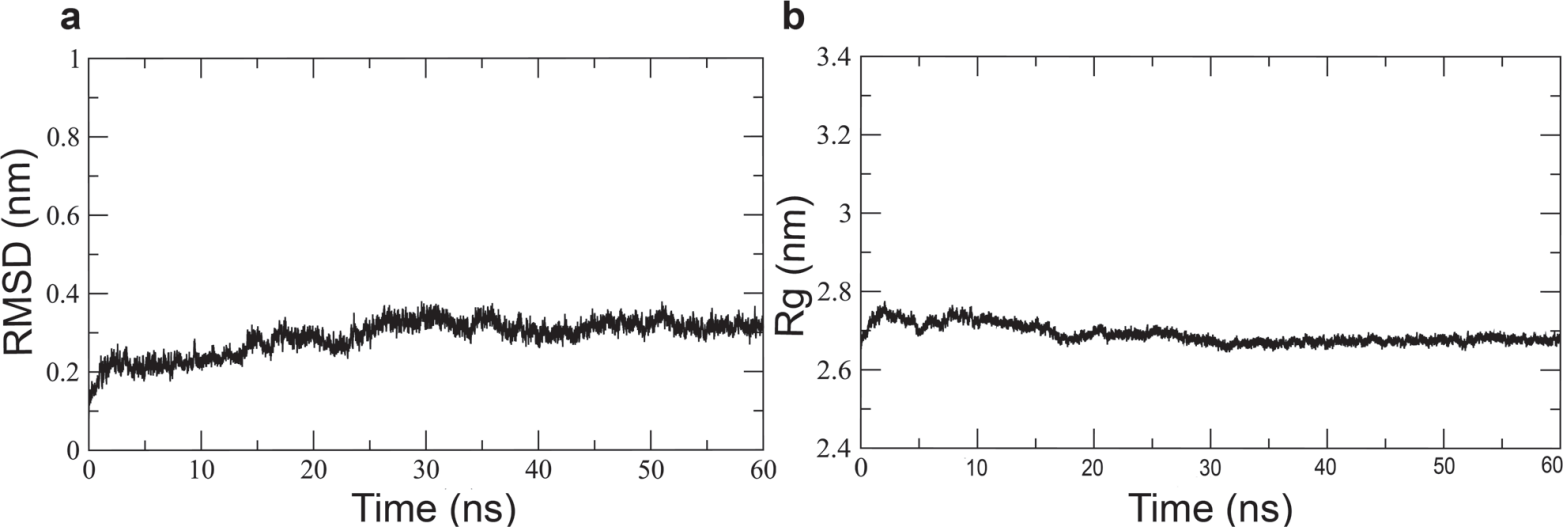
Molecular dynamics results of the DprE1-DprE2 complex. a) RMSD and b) radius of gyration (Rg) of the main chain atoms of DprE1-DprE2 complex over 60 ns of simulation. The figure shows the convergence of the complex throughout the simulation.

**Fig 8:**
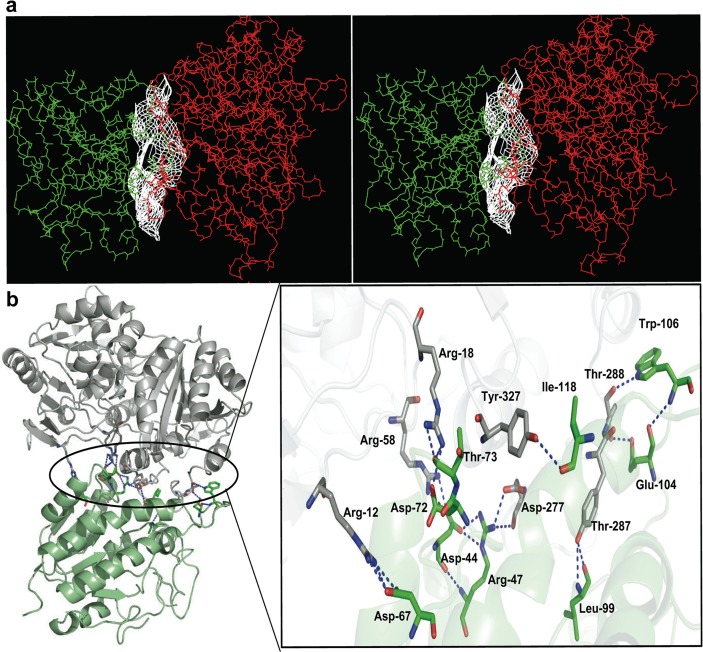
DprE1-DprE2 interaction analysis. (a) The stereo view of the binding surface of the complex where DprE1 and DprE2 are represented by red and green respectively. (b) Cartoon representation of the complex showing interface residues involved in H-bonding where grey and green represents carbon atoms of DprE1 and DprE2 respectively. Oxygen ayoms are coloured red whereas nitrogen atoms are blue. The dashed line represents the H-bond network between residues of DprE1 and DprE2.

The docked complex was minimized to optimize the complex geometry and was subjected to 60 ns MD simulations. The complex was analyzed for its structural integrity in terms of RMSD and Rg which showed that the trajectories of the complex are stable during the simulation (Fig. 7). Protein-protein interaction of lowest metastable structure docking of DprE1 and DprE2, analysis shows that H-bond interaction between residues of DprE1-DprE2 play an important role in the stabilization of the complex. The stereo view of interaction and the residues involved in the H-bonding at the interface of the DprE1-DprE2 complex are shown in Fig. 8 and S1 Movie. The details of the H-bonding, ionic and hydrophobic interactions are summarized in supporting information (S5 and S6 Tables). Hydrophobic DprE1 to DprE2 interactions maintained after MD are Phe-279 to Leu-99, Pro-280 to Leu-99, Leu-283 to Met-197, Tyr-287 to Leu-98, Tyr-287 to Ile-115, Tyr-287 to Val-159, Ile-292 to Trp-106, Leu-295 to Trp-106, Trp-296 to Trp-106, Trp-323 to Ile-118, Tyr-327 to Ile-118 and Tyr-327 to Ala-122. A new hydrophobic interaction is visible between Tyr-287 of DprE1and Phe-158 of DprE2. Only a single hydrogen bonding interaction was maintained after MD and it was observed between Asp-277 of DprE1 and Arg-47 of DprE2. However, at leastfive new hydrogen bonding interactions are formed (DprE1 to DprE2, Gly-282 to Met-197, Tyr-287 to Leu-99, Thr-288 to Trp-106, Thr-288 to Glu-104 and Tyr-327 to Ile-118).The only ionic interaction maintained after MD is between Asp-277 of DprE1 to Arg-47 of DprE2.Interestingly, all the DprE1 residues participating in the interface interaction are dominantly from 272–327 region of DprE1.

**Fig 9:**
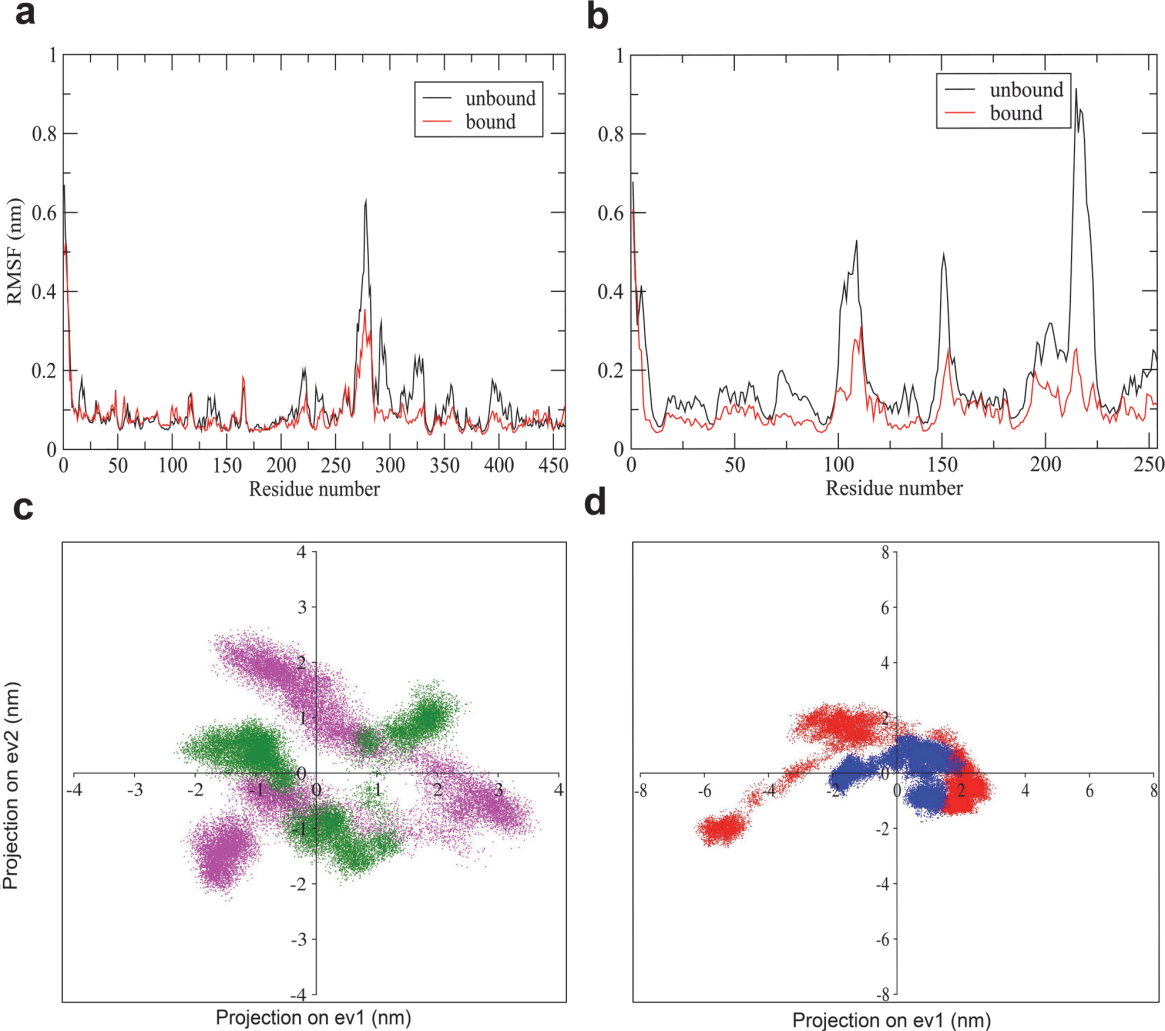
Residue fluctuations and phase space mobility in unbound and bound DprE1 and DprE2. Comparison of the RMSF (Cα atoms) of unbound (black) and bound (red) state of (a) DprE1 and (b) DprE2. Comparison of the projection of the motion of the unbound and bound (c) DprE1 and (d) DprE2 in phase space along the first two eigenvectors. Colours pink and green represents DprE1 in unbound and bound states respectively. The red and blue colour codes for unbound and bound states of DprE2 respectively.

RMSF values of Cα atoms were calculated based on the MD trajectories to explore the structural flexibility in proteins. Fig. 9A and 9B illustrate RMSF value as a function of residue number for both unbound and bound forms. It is clear that the bound DprE1 and DprE2 have lower flexibility as compared to their unbound states. In DprE1 the region 269–330 becomes rigid after the formation of DprE1-DprE2 complex. This region has previously been reported as disordered in the crystal structuresof DprE1. In case of DprE2 high flexibility is observed in the regions 95–113, 146–157 and 197–226 with RMSF >0.4 nm in unbound form.In bound state these regions become rigid as evident from the significant decrease in RMSF value. A dramatic decrease of upto 70% was observed in the region 202–226 for bound DprE2. Our docking results show that the most of the important residues involved in the complexation belong to the region 200–226 (His-200,Leu-201, Glu-203, Leu-206, Thr-207, Asp-209, Tyr-212 and Ala-214) as illustrated in S5–S7 Tables. The results suggest that the region 269–330 of DprE1 and the segment 197–226 of DprE2 are critical for DprE1-DprE2 complexation.

Collective motion of unbound and bound DprE1 and DprE2 was inspected by usingphase space plots as shown in Fig. 9C and 9D. The collective motion provides information about the conformational changes in proteins. We projected MD trajectories on two eigenvectors 1 and 2 for both DprE1 and DprE2. As evident from Fig. 9C, dissimilar eigenvalues were observed in case of unbound and bound DprE1 due to conformational changes.This indicates rich conformational diversity of DprE1. Incase of DprE2, the eigenvalues are similar but significantly reduced implying the bound DprE2 adopts compact structure.

**Fig 10:**
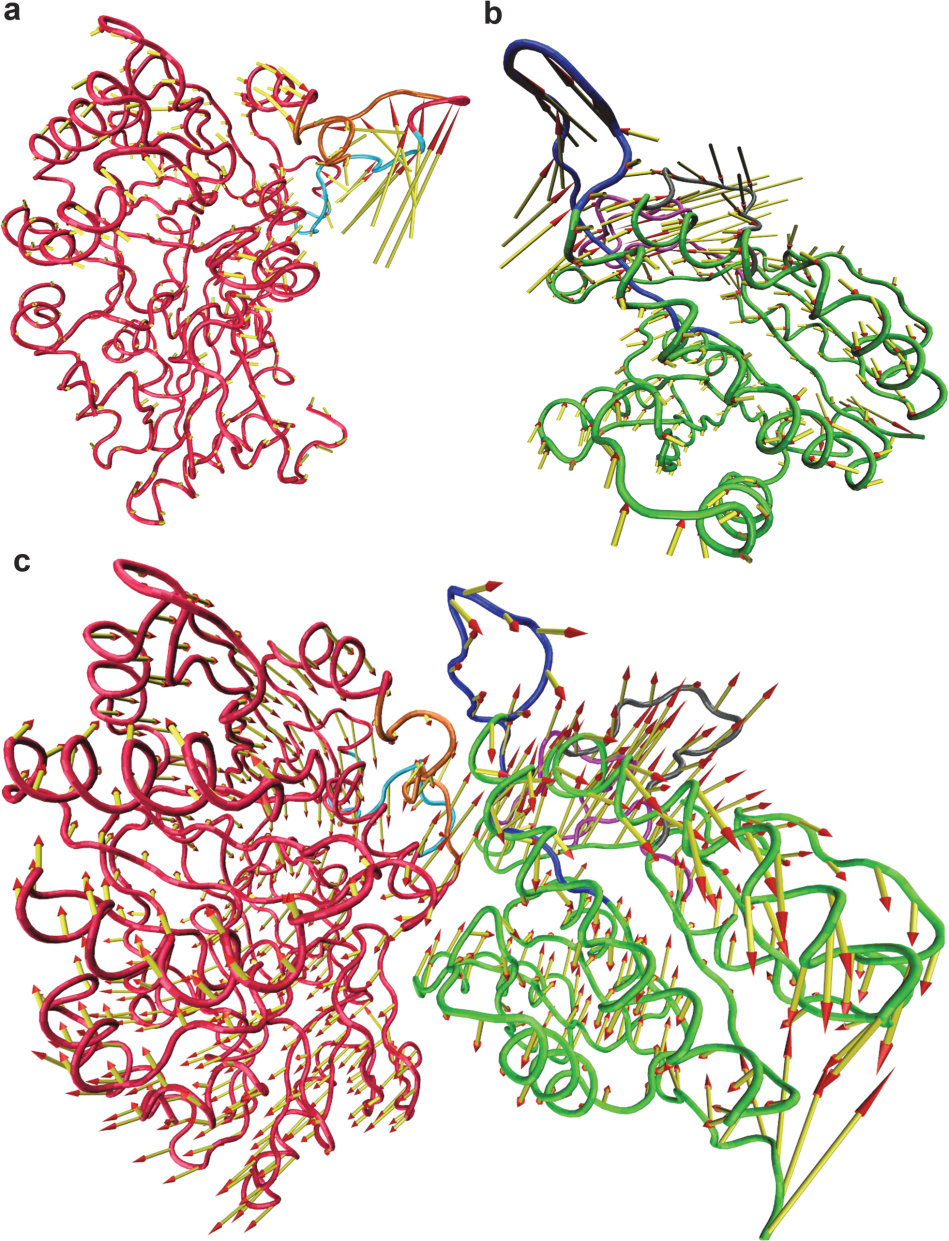
Porcupine plot analysis. The porcupine plot of the first eigenvector of (a) DprE1, (b) DprE2 and (c) DprE1-DprE2 complex. The arrows indicate direction of eignvector and magnitude of the corresponding value. For DprE1, regions 262–275 and 279–291 are shown in cyan and orange respectively. For DprE2, blue, grey and pink represent reions 95–113, 146–157 and 202–226 respectively.

Porcupine describes the subdomain motion of the unbound and bound states of the proteins. Fig. 10 shows the porcupine plots of the topmost eigenvectors of the DprE1, DprE2 and DprE1-DprE2 complex. For DprE1, the most significant conformational changes were observed in the regions encompassing 262–275 and 279–291 residues (Fig. 10A). Upon complexation, both these loopsoccupy the interface of DprE1-DprE2 complex. The loop 262–275 moves upward in the unbound DprE1 whereas in the complex it exhibits a directional change to downward motion. The loop 279–291 showed high intensity upward motion in free DprE1. In complex, loop 279–291 maintained its direction but a significant reduction in magnitude is visible. Also region 276–284 shows a visible concerted motion with residues of DprE2. As a result of these changes, the bound DprE1 exhibits concerted clockwise motion with increase in amplitude.In DprE2, the three regions between the residues 95–113, 146–157 and 202–226 exhibit reduced motion with significant changes in direction (Fig. 10B). In loop 95–113, the motion becomes inward to outward after complex formation. The region 146–157 changed its outward motion in up and outward direction. The loop 202–226 showed an inward motion in unbound DprE2 and an upward motion in bound form. There was some extent of clockwise motion evident after complex formation in DprE2. An interesting observation is the concerted motion of DprE1 residues 276–284 with DprE2 residues at the interface clearly pointing towards complex formation (Fig. 10C). The concerted motions observed in DprE1, DprE2 and at the interface of the DprE1-DprE2 indicate the complex formation give rise to some degree of order within the structures.

**Fig 11:**
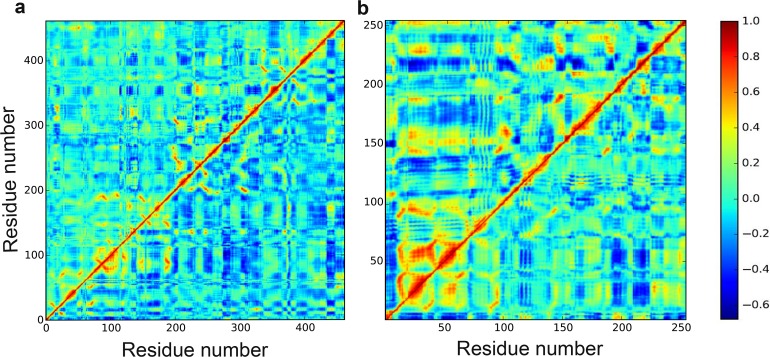
Cross-Correlated motions of unbound and bound DprE1 and DprE2. (a) The correlations for the unbound DprE1 are given in upper left triangle and correlations for bound DprE1 are given in the lower right triangle. (b) The correlations for the unbound DprE2 are given in upper left triangle and correlations for bound DprE2 are given in the lower right triangle. Positive correlations are indicated in red and negative correlations in blue.

Dynamic cross-correlation was conducted to study the interdomain motion of the residues of DprE1 and DprE2 is shown in Fig. 11A and 11B. The value of correlation vary between 1.0 (red) to −0.65 (blue) representing positive and negative correlation respectively. A value of zero (green) implies anti-correlation. For both proteins, the upper left triangle defines the unbound state and the lower right triangle defines the bound state. In the case of DprE1, green color is clearly visible throughout the upper triangle indicating pronounced anti-correlated motion. Interestingly, in bound form green color is less visible and blue is more pronounced implying significant order aftercomplexation with DprE2. A slight positive correlation is observed at region of residues 90–110. In case of DprE2, a dramatic change from positive correlation to negative correlation is observed after complex formation. Most intense positive correlation is between residues10–65where significant changes to anti-correlation after complexation is clear. At region 140–160, a complete transformation from positive to anti-correlation is observed. Regions of residues 90–100, 180–195 and 210–240 demonstrate a major change from positive correlation to anti-correlation. In total, DprE2 exhibit more structural order after complex formation with DprE1.

### MD ensemble-based docking of DprE1 and DprE2

Although the ClusPro docking has been shown to be accurate and reliable in CAPRI experiments [91,92], some studies claim MD based ensemble docking performs better[61,62,63]. It has been shown previously that certain pockets open up impulsively during the MD simulations, some of them might be perfect for binding [93,94]. MD simulations provides various possible conformations of protein to accomplish ensemble-based docking. To perform ensemble docking, we clustered DprE1 and DprE2 MD trajectories into seven clusters each, the first being the most populated whereas the seventh contained the least. The representative structures from all seven clusters for both DprE1 and DprE2 were included in docking. To avoid any bias, each protein conformation of DprE2 was docked into all representative conformations of DprE1, generating a total of 49 complexes. We used the balance scoring function to select the docking poses of these 49 complexes.Based on binding enegies and visual inspection, the top six poses were retrieved from all 49 docking solutions (S5 Fig.). The figure shows that DprE2 shows similar pattern of binding with DprE1 in all the six poses. The interface of DprE1-DprE2 complex is analyzed to check for the residues involved in the interactions. Hydrophobic interactions are observed between the region 272–326 in DprE1 to 95–159 in DprE2. In DprE1, leucine residues at 272, 273 and 275 are the dominant hydrophobic partners. These residues mainly interact either with Val-113 or Trp-106 of DprE2, in all the poses. Dominant ionic interaction moiety of DprE1 is Lys-286 in all the poses. DprE2 region 101–104 consisting of Asp and Glu, interacts with Lys-286 of DprE1. H-bonding segment of DprE1 is in the region 272–292. It interacts with regions 100–110 and 200–210 of DprE2 (S7 Table).

### Contact map and electrostatic analysis

**Fig 12:**
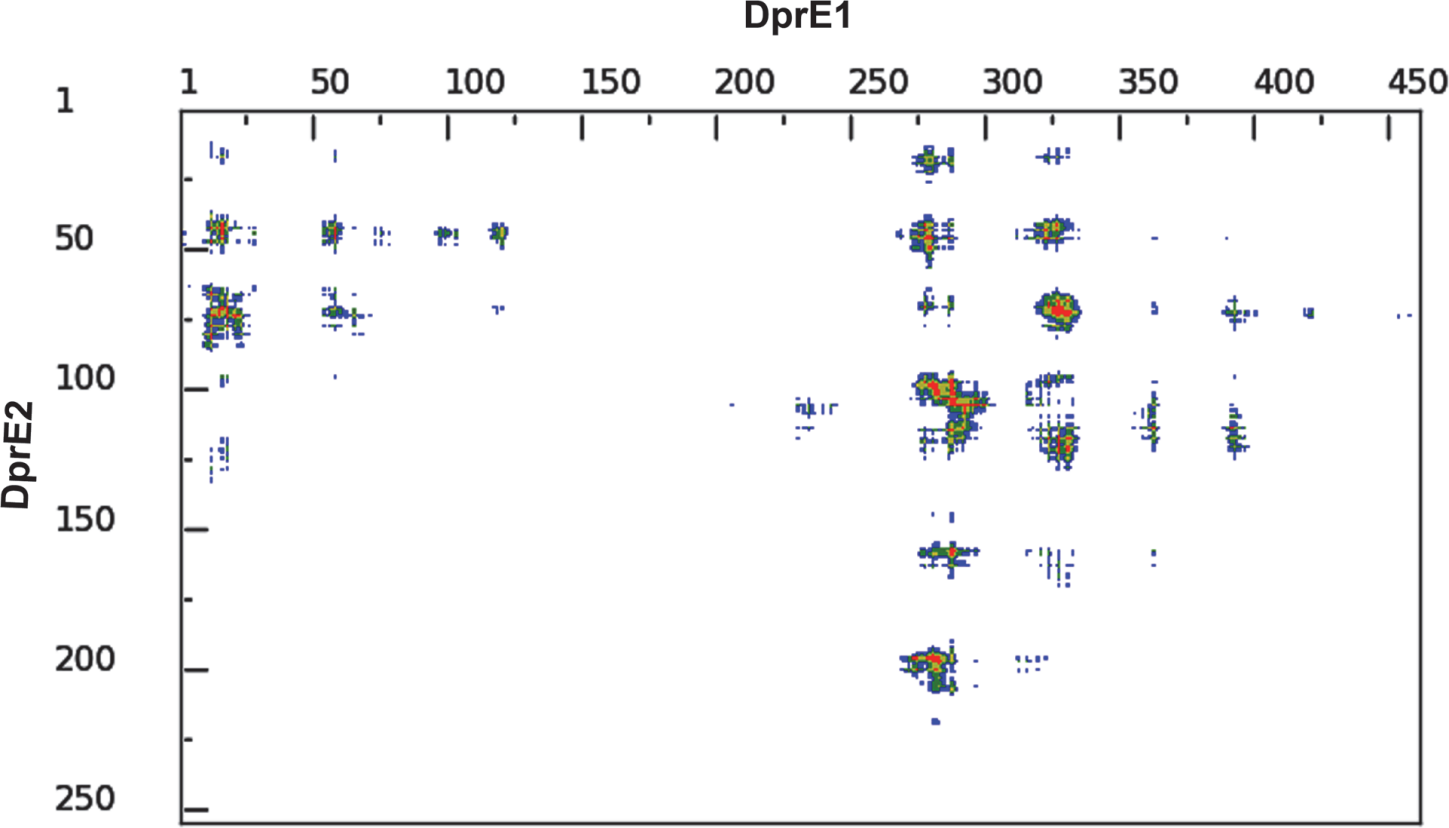
Contact map analysis. The contact map analysis of DprE1 and DprE2. The map shows intermolecular contacts at increasing distances, such that red, yellow, green and blue indicate contacts within 7 Å, 10 Å, 13 Å and 16 Å, respectively.

To illustrate the intramolecular interactions in the DprE1-DprE2 complex, the contact maps were plotted. The distance range intermolecular contact map between the two proteins has been revealed in Fig. 12. The map shows intermolecular contacts at increasing distances, such that red, yellow, green and blue indicate contacts within 7 Å, 10 Å, 13 Å and 16 Å, respectively. As seen in the figure the interface region is mainly the 270–330, a few residues in the region near the N-terminal. On the other hand the interface region in DprE2 seems to be 95–113, 146–157 and 202–226 which correlates with the results obtained through AutoDock and LigPlot+. We also performed the electrostatic potential analysis for the understanding of molecular association of DprE1 and DprE2 (S6 Fig.). Molecular surface electrostatic potential analysis also indicates that the complex is stabilized by electrostatic interactions [95,96].

### Biological aspects of DprE1-DprE2 complex

**Fig 13:**
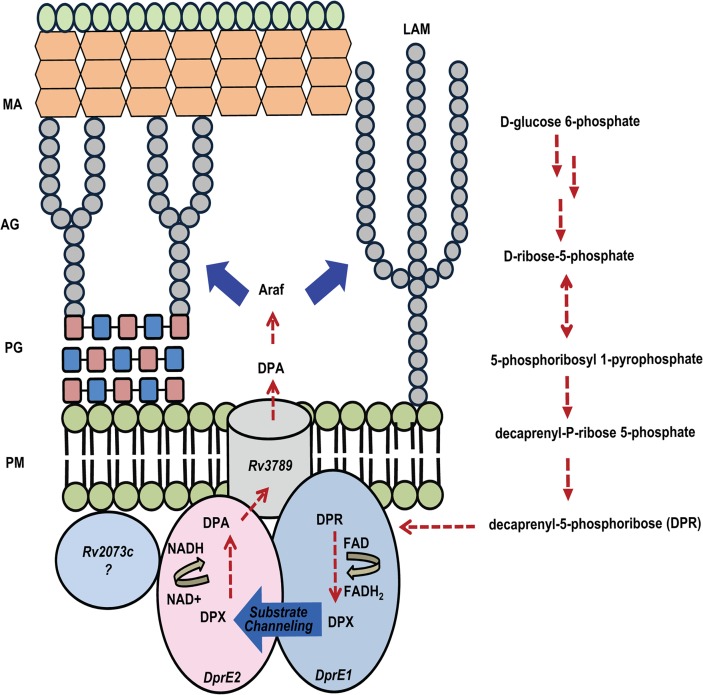
Proposed schematic summary of the biosynthesis of DPA and its translocation to Arabinoglactan synthesis site. The figure shows the various cell wall components MA (Mycolic Acids), AG (Arabinogalactan), PG (Peptidoglycan), PM (Plasma Membrane) and LAM (Lipoarabinomannan). The epimerase complex, DprE1-DprE2 is involved in conversion of DPR to DPA through the intermediate DPX. DPR is synthesized from D-glucose-1-phosphate, through a series of biosynthetic steps. DPA serves as an arabinose donor in the cell wall and is formed by epimerization by DprE1 (Rv3790) and DprE2 (Rv3791). Rv2073c is a short chain dehydrogenase (249 residues) which shows interaction with DprE2.

Our results indicate that DprE1-DprE2 form epimerase complex and possibly plays a major role in the catalysis of DPR to DPA. Disrupting DprE1-DprE2 interaction with small molecules or peptides might be a potential therapeutic target. DPA is the precursor of Araf which subsequently incorporates into the AG and LAM layers of bacterial cell wall. Fig. 13 represents the scheme for the biosynthesis of DPA. In mycobacteria, DPA biosynthesis takes place at the plasma membrane [97] through a pathway initiating from D-glucose-6-phosphate [17]. DPR is the substrate for DprE1 and is oxidized to DPX. The intermediate DPX is then transferred to DprE2 subunit of the epimerase complex, most probably through a ‘*substrate channel*’ at the interface of DprE1-DprE2 complex. The NAD^+^ dependent reduction of DPX leads to the formation of DPA. A translocase enzyme, Rv3789 is located upstream of the DprE1-DprE2 complex and is probably involved in the reorientation and export of DPA across the plasma membrane to the site of AG and LAM synthesis [98]. In *M*. *smegmatis*, redundancy of DprE2 has been observed to Rv2073c, a short chain dehydrogenase (249 residues), containing NAD(P) binding domain, which acts as a catalytic substitute. As the essentiality of DprE2 has already been demonstrated in *Mtb* [24].Thus, DprE1-DprE2 complex could be a potential drug target for pharmacological intervention.

## Conclusion and Future Prospects

We have analyzed and characterized the structural and dynamic features of DprE1, DprE2, and the DprE1-DprE2 complex using homology modeling, protein threading, molecular dynamics studies and the available crystal structures of DprE1. Homology modeling and simulation studies provided missing structural information on the disorder regions in the crystal structure of DprE1. We generated three-dimensional models of DprE2 using a protein threading approach. The DprE2 structure shows the folds characteristic of the reductase protein family. Molecular dynamics studies performed over 50 ns showed that the structures of both DprE1 and DprE2 are stable over the course of the simulation. The convergence of sampling in both *Mtb* DprE1 and *Mtb* DprE2 was achieved. PCA analysis suggested that in the case of *Mtb* DprE1, residues from 269–330 show greater fluctuation, which agrees well with the reported crystal structure in which this region is disordered. In the case of DprE2, large fluctuations were observed in residues 95–113, 146–157, and 197–226. The data indicate that both DprE1 and DprE2 are able to utilize diverse conformational space. The DprE1-DprE2 docked complex revealed the key interacting residues. Ensemble based docking analysis of DprE1-BTZ043 showed that the ligand is stabilized by hydrogen bonding interaction with the Lys-134, Lys-367, Gln-336, Cys-387residues of DprE1. The study sheds light on mechanistic aspects of DPA biosynthesis and would also prove useful in the development of novel therapeutics against TB. Further work will focus on experimental investigations to determine the molecular basis for substrate recognition and catalysis of DprE1/DprE2 exploring the detailed mechanistic studies.

## Supporting Information

S1 Fig Ramachandran plot of the DprE1 homology model. The most favored regions are red. Additionally allowed, generously allowed, and disallowed regions are indicated as yellow, light yellow, and white, respectively. No residue lies in the disallowed region, indicating high stereochemical quality.
(TIF)

S2 Fig Ramachandran plot of the three dimensional DprE2 model. The most favored regions are red. Additionally allowed, generously allowed, and disallowed regions are indicated as yellow, light yellow, and white, respectively. Figure shows no stereochemical clashes in the generated model.
(TIF)

S3 Fig Secondary structure consensus of *Mtb* DprE2. The consensus was built manually by tools such as YASPIN, Scratch, GOR4, PSIPRED and PSSpred.
(TIF)

S4 Fig Superimposition of the top DprE1-DprE2 complexes by three independent servers. Grey, green and purple represents the top complexes by ClusPro, GRAMM-X and PatchDock respectively.
(TIF)

S5 Fig Superimposition of top six poses as obtained from ensemble based docking. DprE1 and DprE2 are shown in grey and green colors respectively.
(TIF)

S6 Fig Surface electrostatic potential of a) DprE1 and b) DprE2 illustrating the charge distribution of molecules, hence showing molecular association. Red and blue indicate negative and positive regions respectively.
(TIF)

S1 Movie The movie shows the transitions in the DprE1-DprE2 trajectory over the course of 60 ns. The DprE1and DprE2 are shown in ribbon representation with yellow and green colour respectively. The residues involved in hydrogen bonding are labeled.
(ZIP)

S1 Table The What-if “fine check quality control” analysis of (a) DprE1 (b) DprE2 before and after the refined model. (DOCX)

S2 Table Validation of DprE1 and DprE2 generated models. (DOCX)

S3 Table Binding energies of docked DprE1-BTZ043 complex. (DOCX)

S4 Table Comparison of *Mtb* DprE1-BTZ043 binding interactions with other reported DprE1 inhibitors. (DOCX)

S5 Table Hydrogen bonding interactions involved at the interface between DprE1-DprE2 complex (DOCX)

S6 Table Hydrophobic and ionic interactions involved in DprE1-DprE2 complex formation. (DOCX)

S7 Table Ensemble-based docking:the binding site interactions of DprE1-DprE2 complex from top six poses (a-f). (DOCX)
