# Supplementary figures and images for: Structure, Dynamics, and Interaction of Mycobacterium tuberculosis (Mtb) DprE1 and DprE2 Examined by Molecular Modeling, Simulation, and Electrostatic Studies
Source: PLoS One. 2015 Mar 19;10(3):e0119771. doi: 10.1371/journal.pone.0119771 (PMC4366402; doi:10.1371/journal.pone.0119771)

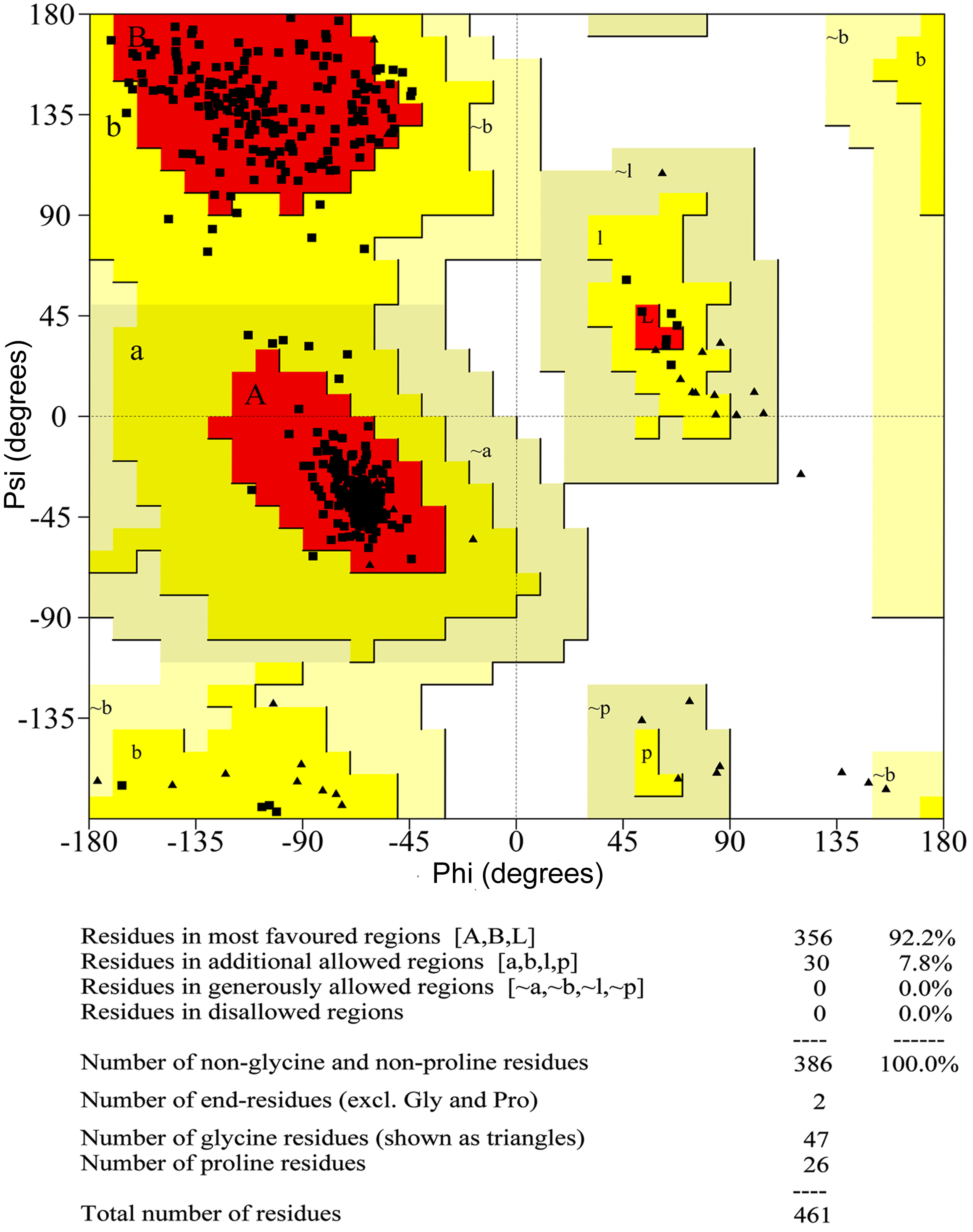

Supplement: S1 Fig — The most favored regions are red. Additionally allowed, generously allowed, and disallowed regions are indicated as yellow, light yellow, and white, respectively. No residue lies in the disallowed region, indicating high stereochemical quality. (TIF) [file pone.0119771.s001.tif]

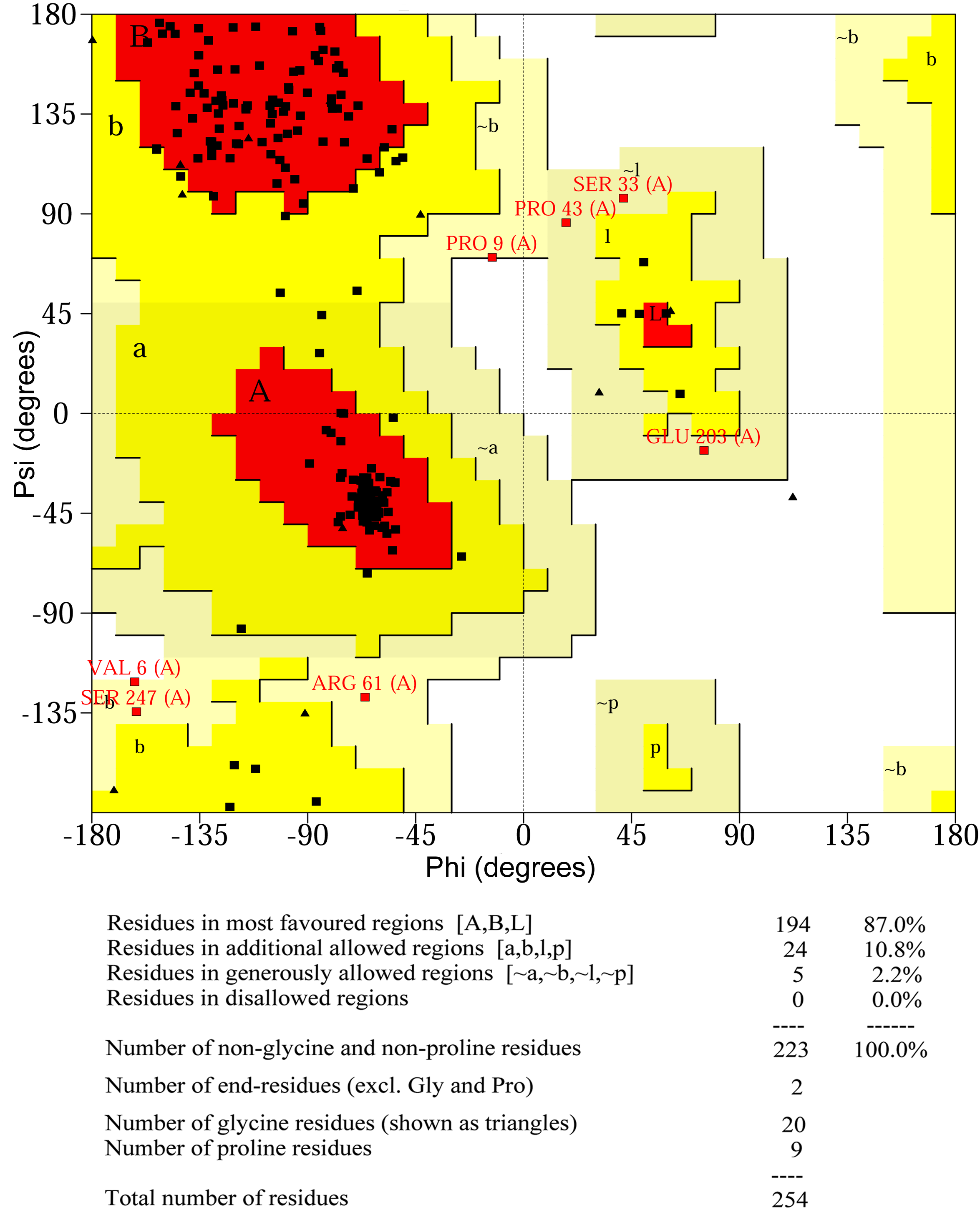

Supplement: S2 Fig — The most favored regions are red. Additionally allowed, generously allowed, and disallowed regions are indicated as yellow, light yellow, and white, respectively. Figure shows no stereochemical clashes in the generated model. (TIF) [file pone.0119771.s002.tif]

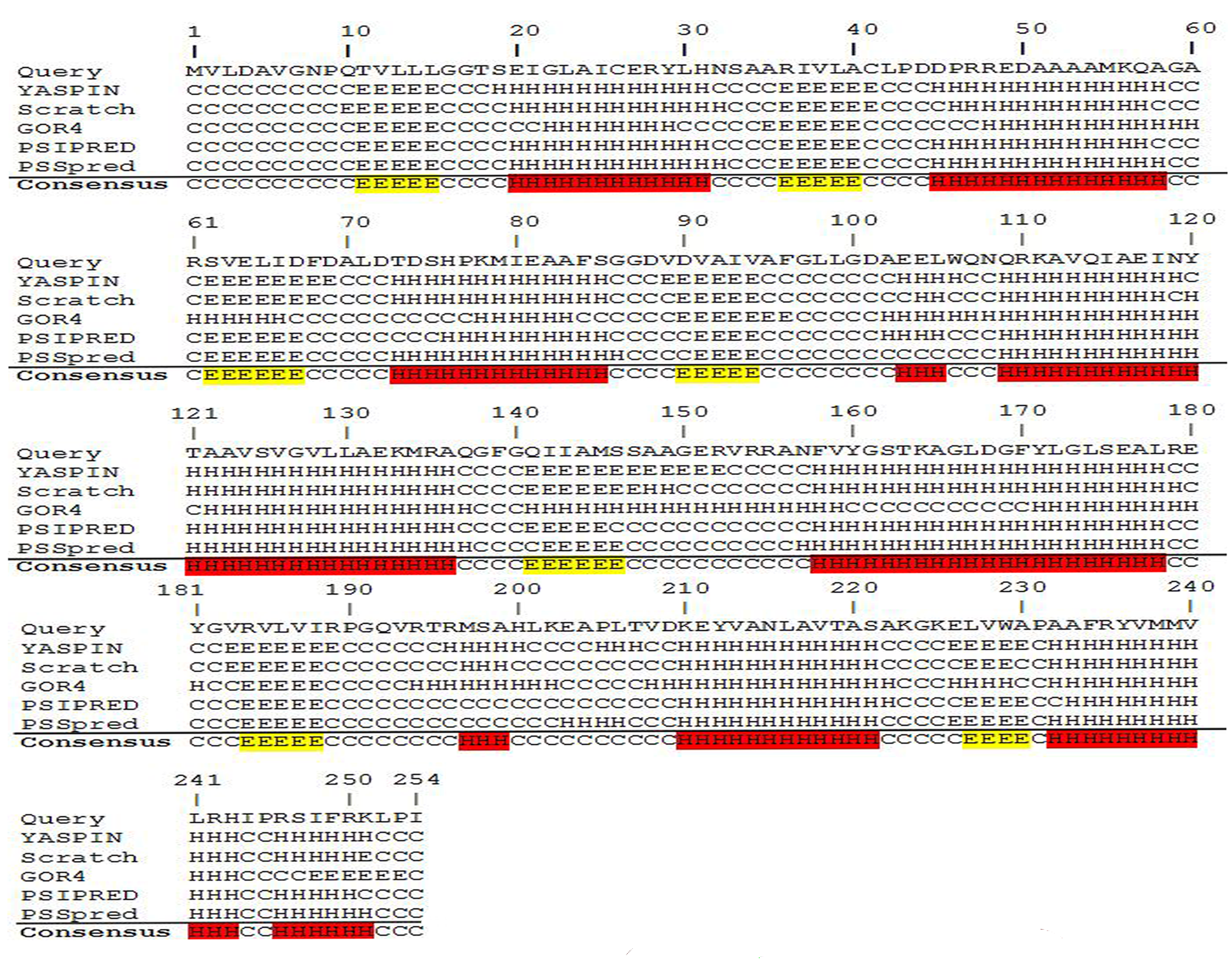

Supplement: S3 Fig — The consensus was built manually by tools such as YASPIN, Scratch, GOR4, PSIPRED and PSSpred. (TIF) [file pone.0119771.s003.tif]

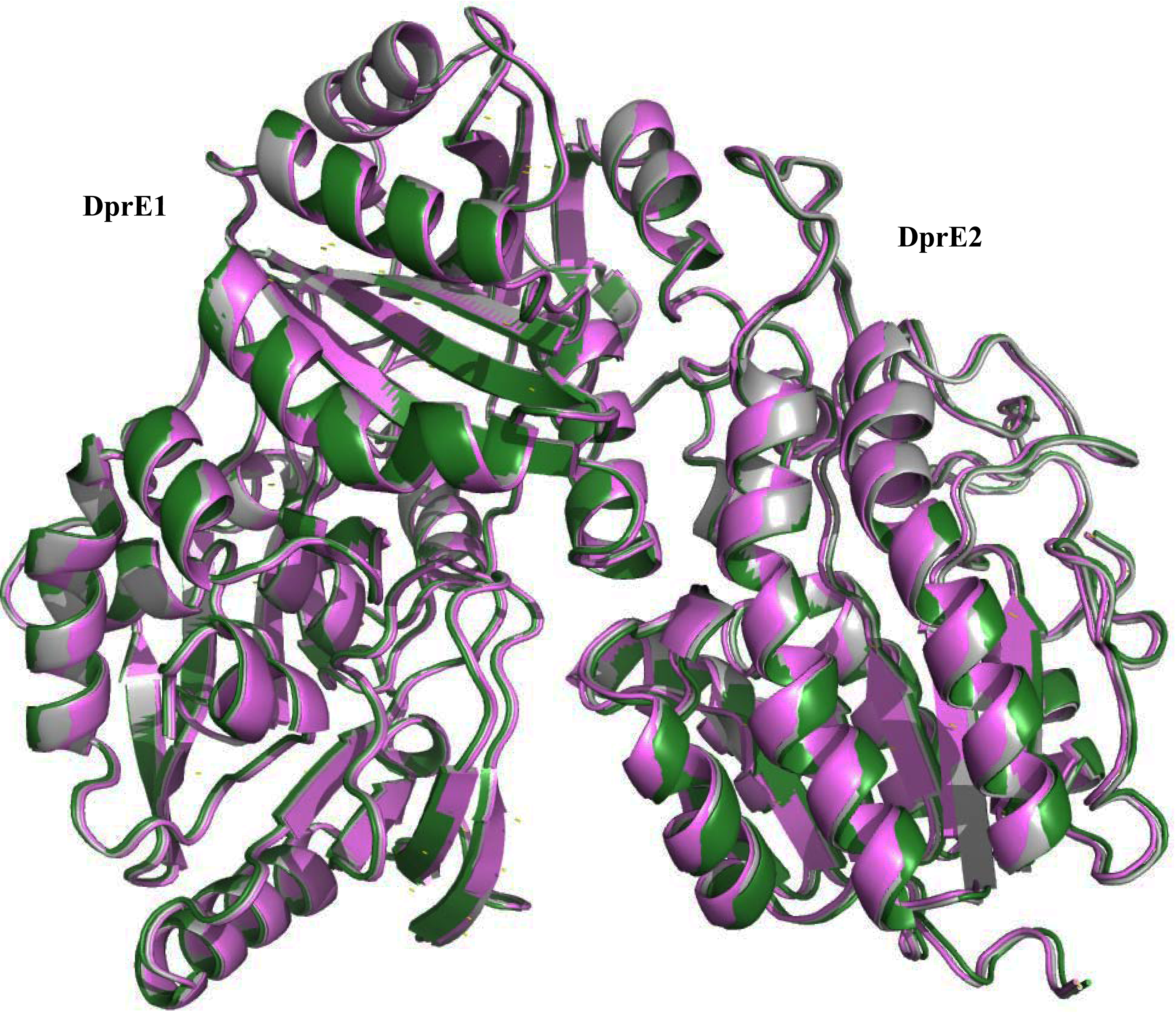

Supplement: S4 Fig — Grey, green and purple represents the top complexes by ClusPro, GRAMM-X and PatchDock respectively. (TIF) [file pone.0119771.s004.tif]

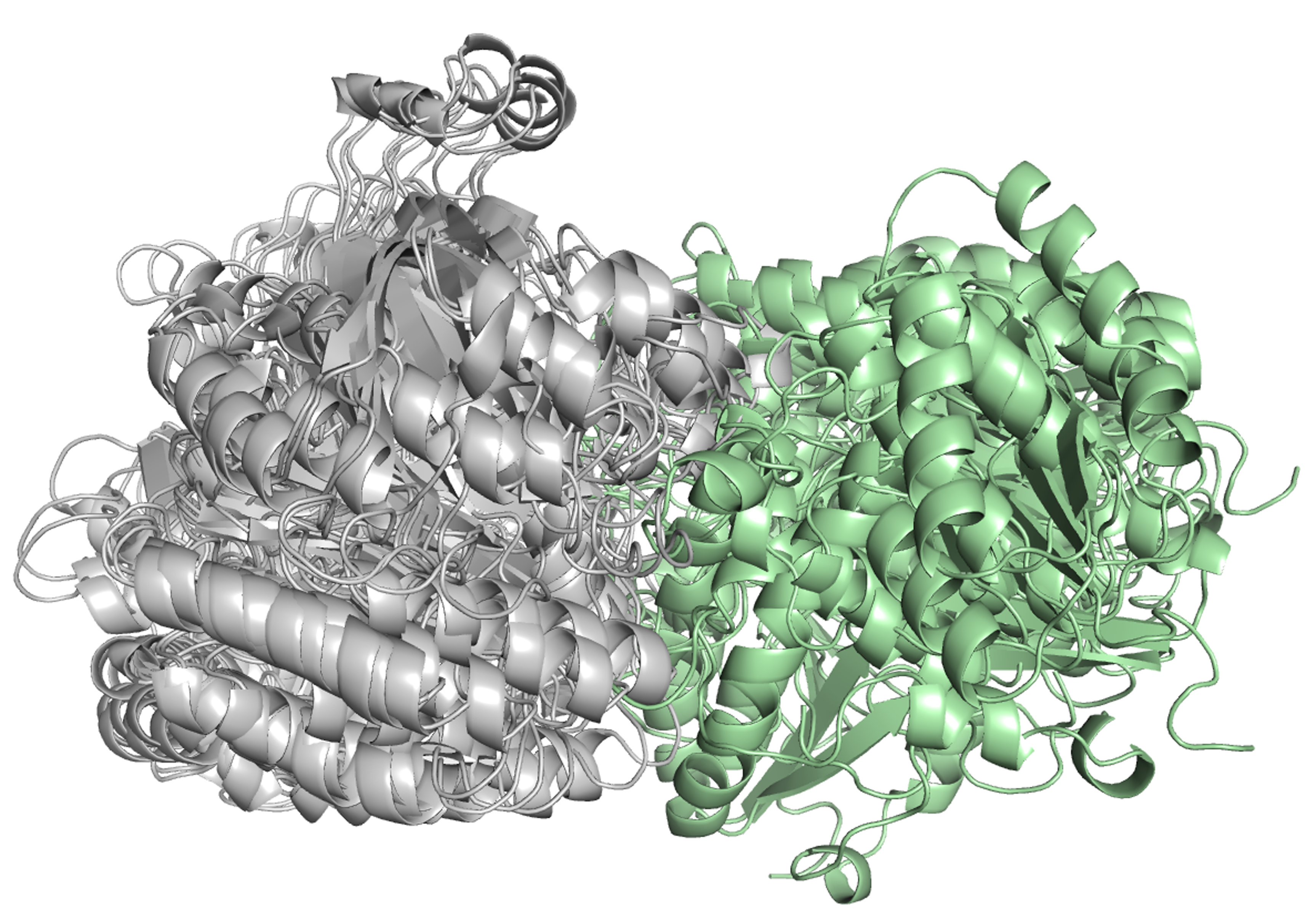

Supplement: S5 Fig — DprE1 and DprE2 are shown in grey and green colors respectively. (TIF) [file pone.0119771.s005.tif]

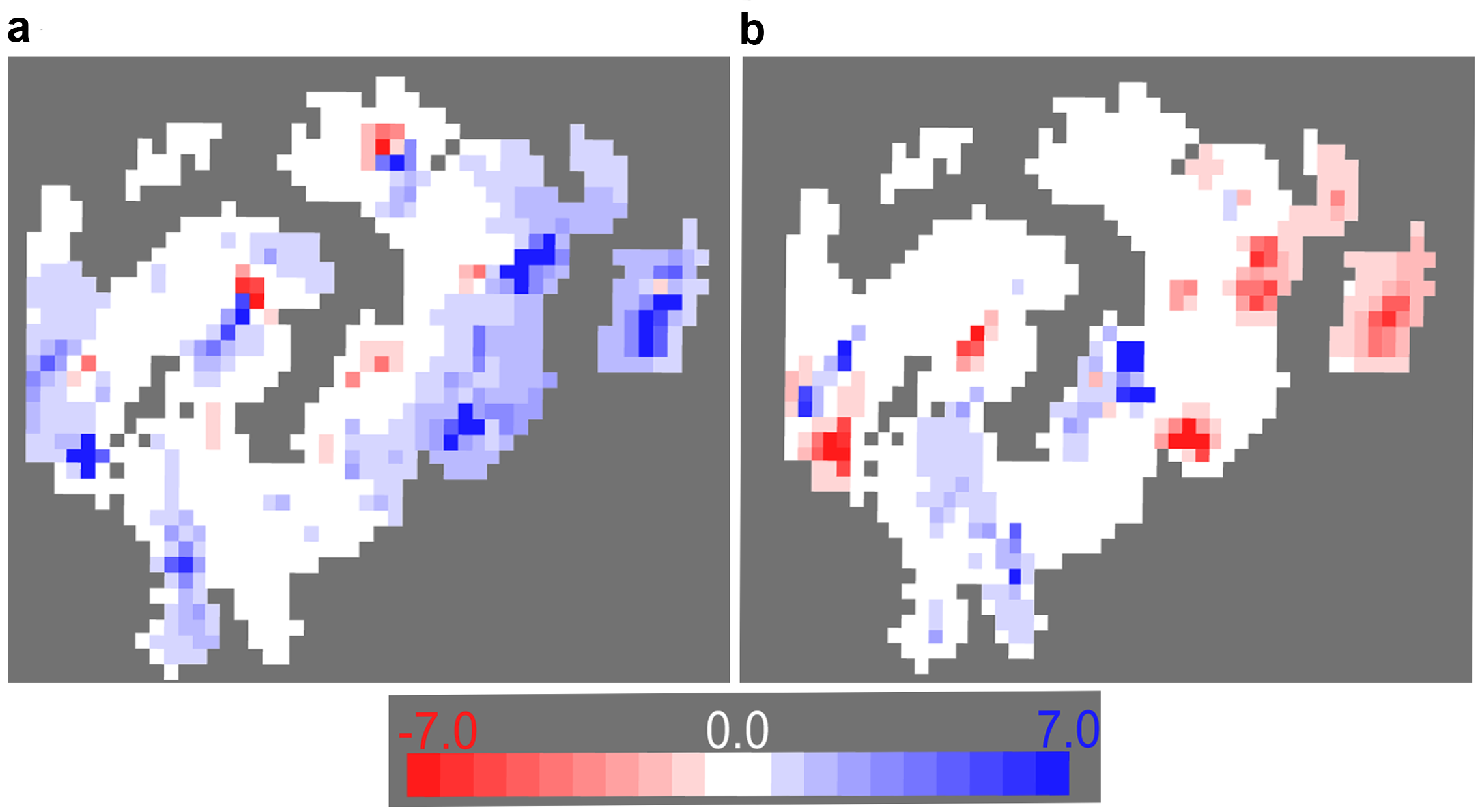

Supplement: S6 Fig — Red and blue indicate negative and positive regions respectively. (TIF) [file pone.0119771.s006.tif]
