# Supplementary material for: Structure, Dynamics, and Interaction of Mycobacterium tuberculosis (Mtb) DprE1 and DprE2 Examined by Molecular Modeling, Simulation, and Electrostatic Studies
Source: PLoS One. 2015 Mar 19;10(3):e0119771. doi: 10.1371/journal.pone.0119771 (PMC4366402; doi:10.1371/journal.pone.0119771)
Supplement: S1 Table — (DOCX) [file pone.0119771.s008.docx]

**Table S1. The What-if “fine check quality control” analysis of (a) DprE1 (b) DprE2 before and after the refined model.**

**a.**

| **DprE1 models** | **Backbone-backbone contacts** | **Backbone-side chain contacts** | **Side chain-backbone contacts** | **Side chain-side chain contacts** |
| --- | --- | --- | --- | --- |
| Initial Model (Before MD) | -1.01 | -1.98 | -1.41 | -1.23 |
| Refined model  (Afetr MD) | -1.78 | -1.57 | -1.89 | -1.29 |

**b.**

| **DprE2 Models** | **Backbone-backbone contacts** | **Backbone-side chain contacts** | **Side chain-backbone contacts** | **Side chain-side chain contacts** |
| --- | --- | --- | --- | --- |
| Initial Model (Before MD) | -2.05 | -2.77 | -2.97 | -1.82 |
| Refined model  (Afetr MD) | -2.11 | -2.09 | -1.20 | -0.83 |
