# Supplementary material for: Structure, Dynamics, and Interaction of Mycobacterium tuberculosis (Mtb) DprE1 and DprE2 Examined by Molecular Modeling, Simulation, and Electrostatic Studies
Source: PLoS One. 2015 Mar 19;10(3):e0119771. doi: 10.1371/journal.pone.0119771 (PMC4366402; doi:10.1371/journal.pone.0119771)
Supplement: S2 Table — (DOCX) [file pone.0119771.s009.docx]

**Table S2.** **Validation of DprE1 and DprE2 generated models.**

| **S.No** | **Tools** | **DprE1** | **DprE2** |
| --- | --- | --- | --- |
| 1. | ERRAT^a^ | 90.90% | 75.20% |
| 2. | ProQ^b^ | LG score: 4.508; MaxSub: 0.282 | LG score: 3.38; Maxsub: 2.64 |
| 3. | SolvX^c^ | -94.7 | -38.9 |

^a^The normally-accepted range is > 50 for a high quality model.

^b^The ProQ LG score > 1.5 and MaxSub score > 0.1 indicates a fine quality model.

^c^The SolvX profile score < 0 is considered a well packed model.
