# Supplementary material for: Structure, Dynamics, and Interaction of Mycobacterium tuberculosis (Mtb) DprE1 and DprE2 Examined by Molecular Modeling, Simulation, and Electrostatic Studies
Source: PLoS One. 2015 Mar 19;10(3):e0119771. doi: 10.1371/journal.pone.0119771 (PMC4366402; doi:10.1371/journal.pone.0119771)
Supplement: S3 Table — (DOCX) [file pone.0119771.s010.docx]

**Table S3.** **Binding energies of docked DprE1-BTZ043 complex.**

| **BTZ043-DprE1 complex** | **Binding Energy (kcal/mol)** |
| --- | --- |
| Complex 1 | -9.25 |
| Complex 2 | -9.84 |
| Complex 3 | -11.04 |
| Complex 4 | -7.72 |
| Complex 5 | -11.49 |
| Complex 6 | -10.14 |
