# Supplementary material for: Structure, Dynamics, and Interaction of Mycobacterium tuberculosis (Mtb) DprE1 and DprE2 Examined by Molecular Modeling, Simulation, and Electrostatic Studies
Source: PLoS One. 2015 Mar 19;10(3):e0119771. doi: 10.1371/journal.pone.0119771 (PMC4366402; doi:10.1371/journal.pone.0119771)
Supplement: S4 Table — (DOCX) [file pone.0119771.s011.docx]

**Table S4. Comparison of *Mtb* DprE1-BTZ043 binding interactions with other reported DprE1 inhibitors.**

| **Protein** | **Inhibitor** | **Binding Interactions** | **Binding Interactions common to DprE1-BTZ043** | **Unique binding interactions with *(M.tuberculosis)*** **DprE1-BTZ043** |
| --- | --- | --- | --- | --- |
| **DprE1 *(M.smegmatis)*** | BTZ046 | Tyr-67, His-139, Gly-140, Lys-141, Leu-324, Phe-327, Gln-341, Gln-343, Leu-370, Val-372, Lys-374, Phe-376, Asn-392, Cys-394, Lys-425 | Tyr-67, Gly-140, Lys-141, Gln-334, Gln-336, Lys-374, Asn-392, Cys-394, Lys-425 (corresponding to Tyr-60, Gly-133, Lys-134, Gln-327, Gln-329, Lys-367, Asn-385, Cys-387, Lys-418 in *Mtb* respectively) | His-139, Leu-317, Phe-327, Leu-370, Val-372, Phe-376 (corresponding to His-132, Leu-310, Phe-320, Leu-363, Val-365, Phe-369 in *Mtb* respectively) |
| **DprE1 *(M.tuberculosis)*** | CT325 | Tyr-60, Gly-117, His-132, Gly-133, Lys-134, Ser-228, Trp-230, Gln-336, Val-365, Lys-367, Asn-385, Cys-387. | Tyr-60, Gly-133, Lys-134, Gln-336, Lys-367, Asn-385, Cys-387. | Gly-117, His-132, Ser-228, Trp-230, Val-365 |
| **DprE1 *(M.tuberculosis)*** | TCA1 | Tyr-60, His-132, Lys-134, Ser-228, Trp-230, Tyr-314, Gln-334, Gln-336, Val-365, Lys-367, Asn-385, Cys-387, Lys-418. | Tyr-60, Lys-134, Tyr-314, Gln-334, Gln-336, Lys-367, Asn-385, Cys-387, Lys-418. | His-132, Ser-228, Trp-230, Val-365 |
| **DprE1 *(M.tuberculosis)*** | PBTZ169 | Leu-115, Gly-117, Trp-230, Leu-363, Val-365, Cys-387, Lys-418 | Cys-387, Lys-418 | Leu-115, Gly-117, Trp-230, Leu-363, Val-365 |
