# Supplementary material for: Structure, Dynamics, and Interaction of Mycobacterium tuberculosis (Mtb) DprE1 and DprE2 Examined by Molecular Modeling, Simulation, and Electrostatic Studies
Source: PLoS One. 2015 Mar 19;10(3):e0119771. doi: 10.1371/journal.pone.0119771 (PMC4366402; doi:10.1371/journal.pone.0119771)
Supplement: S5 Table — (DOCX) [file pone.0119771.s012.docx]

**Table S5.** **Hydrogen bonding interactions involved at the interface between DprE1-DprE2 complex**

| **Molecule** | **Residue number** | **Residue code** | **Molecule** | **Residue number** | **Residue code** |
| --- | --- | --- | --- | --- | --- |
| DprE1 | 12 | Arg | DprE2 | 67 | Asp |
| DprE1 | 18 | Arg | DprE2 | 72 | Asp |
| DprE1 | 18 | Arg | DprE2 | 73 | Thr |
| DprE1 | 58 | Arg | DprE2 | 44 | Asp |
| DprE1 | 277 | Asp | DprE2 | 47 | Arg |
| DprE1 | 287 | Tyr | DprE2 | 99 | Leu |
| DprE1 | 288 | Thr | DprE2 | 106 | Trp |
| DprE1 | 288 | Thr | DprE2 | 104 | Glu |
| DprE1 | 327 | Tyr | DprE2 | 118 | Leu |
