# Supplementary material for: Structure, Dynamics, and Interaction of Mycobacterium tuberculosis (Mtb) DprE1 and DprE2 Examined by Molecular Modeling, Simulation, and Electrostatic Studies
Source: PLoS One. 2015 Mar 19;10(3):e0119771. doi: 10.1371/journal.pone.0119771 (PMC4366402; doi:10.1371/journal.pone.0119771)
Supplement: S6 Table — (DOCX) [file pone.0119771.s013.docx]

**Table S6.** **Hydrophobic and ionic interactions involved in DprE1-DprE2 complex formation**.

| **Molecule** | **Residue number** | **Residue code** | **Molecule** | **Residue number** | **Residue code** |
| --- | --- | --- | --- | --- | --- |
| **Hydrophobic Interactions** | | | | | |
| DprE1 | 16 | Trp | DprE2 | 43 | Pro |
| DprE1 | 278 | Val | DprE2 | 46 | Pro |
| DprE1 | 280 | Pro | DprE2 | 99 | Leu |
| DprE1 | 283 | Leu | DprE2 | 197 | Met |
| DprE1 | 287 | Tyr | DprE2 | 98 | Leu |
| DprE1 | 287 | Tyr | DprE2 | 115 | Ile |
| DprE1 | 287 | Tyr | DprE2 | 158 | Phe |
| DprE1 | 287 | Tyr | DprE2 | 159 | Val |
| DprE1 | 292 | Ile | DprE2 | 106 | Trp |
| DprE1 | 295 | Leu | DprE2 | 106 | Trp |
| DprE1 | 296 | Trp | DprE2 | 106 | Trp |
| DprE1 | 323 | Tyr | DprE2 | 118 | Ile |
| DprE1 | 327 | Tyr | DprE2 | 118 | Ile |
| DprE1 | 327 | Tyr | DprE2 | 122 | Ala |
| **Ionic interactions** | | | | | |
| DprE1 | 12 | Arg | DprE2 | 67 | Asp |
| DprE1 | 12 | Arg | DprE2 | 81 | Glu |
| DprE1 | 18 | Arg | DprE2 | 72 | Asp |
| DprE1 | 18 | Arg | DprE2 | 74 | Asp |
| DprE1 | 58 | Arg | DprE2 | 44 | Asp |
| DprE1 | 277 | Asp | DprE2 | 47 | Arg |
| DprE1 | 287 | Tyr | DprE2 | 158 | Phe |
| DprE1 | 287 | Tyr | DprE2 | 159 | Val |
