# Supplementary material for: Structure, Dynamics, and Interaction of Mycobacterium tuberculosis (Mtb) DprE1 and DprE2 Examined by Molecular Modeling, Simulation, and Electrostatic Studies
Source: PLoS One. 2015 Mar 19;10(3):e0119771. doi: 10.1371/journal.pone.0119771 (PMC4366402; doi:10.1371/journal.pone.0119771)
Supplement: S7 Table — (DOCX) [file pone.0119771.s014.docx]

**Table S7. Ensemble based docking: the binding site interactions of DprE1-DprE2 complex from top six poses (a-f).**

**a.**

| **Molecule** | **Residue number** | **Residue code** | **Molecule** | **Residue number** | **Residue code** |
| --- | --- | --- | --- | --- | --- |
| **Hydrophobic Interactions** | | | | | |
| DprE1 | 234 | Ile | DprE2 | 46 | Pro |
| DprE1 | 272 | Leu | DprE2 | 106 | Trp |
| DprE1 | 283 | Leu | DprE2 | 112 | Ala |
| DprE1 | 283 | Leu | DprE2 | 115 | Ile |
| DprE1 | 283 | Leu | DprE2 | 158 | Phe |
| DprE1 | 283 | Leu | DprE2 | 159 | Val |
| DprE1 | 287 | Tyr | DprE2 | 96 | Phe |
| DprE1 | 287 | Tyr | DprE2 | 98 | Leu |
| DprE1 | 287 | Tyr | DprE2 | 115 | Ile |
| DprE1 | 287 | Tyr | DprE2 | 118 | Ile |
| DprE1 | 292 | Ile | DprE2 | 118 | Ile |
| DprE1 | 295 | Leu | DprE2 | 43 | Pro |
| DprE1 | 326 | Ala | DprE2 | 206 | Leu |
| DprE1 | 327 | Tyr | DprE2 | 197 | Met |
| DprE1 | 360 | Tyr | DprE2 | 46 | Pro |
| **Ionic interactions** | | | | | |
| DprE1 | 242 | Arg | DprE2 | 72 | Asp |
| DprE1 | 286 | Lys | DprE2 | 101 | Asp |
| DprE1 | 286 | Lys | DprE2 | 103 | Glu |
| DprE1 | 298 | Arg | DprE2 | 45 | Asp |
| DprE1 | 298 | Arg | DprE2 | 72 | Asp |
| **Hydrogen bonding** | | | | | |
| DprE1 | 18 | Arg | DprE2 | 205 | Pro |
| DprE1 | 271 | Gln | DprE2 | 104 | Glu |
| DprE1 | 281 | Asn | DprE2 | 107 | Gln |
| DprE1 | 283 | Leu | DprE2 | 104 | Glu |
| DprE1 | 283 | Leu | DprE2 | 110 | Arg |
| DprE1 | 298 | Arg | DprE2 | 72 | Asp |
| DprE1 | 325 | Arg | DprE2 | 206 | Leu |
| DprE1 | 326 | Ala | DprE2 | 207 | Thr |

**b.**

| **Molecule** | **Residue number** | **Residue code** | **Molecule** | **Residue number** | **Residue code** |
| --- | --- | --- | --- | --- | --- |
| **Hydrophobic Interactions** | | | | | |
| DprE1 | 16 | Trp | DprE2 | 105 | Leu |
| DprE1 | 272 | Leu | DprE2 | 113 | Val |
| DprE1 | 273 | Leu | DprE2 | 106 | Trp |
| DprE1 | 275 | Leu | DprE2 | 106 | Trp |
| DprE1 | 278 | Val | DprE2 | 112 | Ala |
| DprE1 | 278 | Val | DprE2 | 115 | Ile |
| DprE1 | 278 | Val | DprE2 | 158 | Phe |
| DprE1 | 278 | Val | DprE2 | 159 | Val |
| DprE1 | 279 | Phe | DprE2 | 113 | Val |
| **Ionic interactions** | | | | | |
| DprE1 | 12 | Arg | DprE2 | 203 | Glu |
| DprE1 | 18 | Arg | DprE2 | 209 | Asp |
| DprE1 | 58 | Arg | DprE2 | 209 | Asp |
| DprE1 | 286 | Lys | DprE2 | 101 | Asp |
| DprE1 | 286 | Lys | DprE2 | 104 | Glu |
| DprE1 | 325 | Arg | DprE2 | 103 | Glu |
| **Hydrogen bonding** | | | | | |
| DprE1 | 12 | Arg | DprE2 | 203 | Glu |
| DprE1 | 18 | Arg | DprE2 | 205 | Pro |
| DprE1 | 18 | Arg | DprE2 | 206 | Leu |
| DprE1 | 18 | Arg | DprE2 | 209 | Asp |
| DprE1 | 54 | Arg | DprE2 | 212 | Tyr |
| DprE1 | 277 | Asp | DprE2 | 101 | Asp |
| DprE1 | 277 | Asp | DprE2 | 102 | Ala |
| DprE1 | 277 | Asp | DprE2 | 157 | Asn |
| DprE1 | 285 | Asp | DprE2 | 104 | Glu |
| DprE1 | 286 | Lys | DprE2 | 104 | Glu |
| DprE1 | 322 | Glu | DprE2 | 103 | Glu |

**c.**

| **Molecule** | **Residue number** | **Residue code** | **Molecule** | **Residue number** | **Residue code** |
| --- | --- | --- | --- | --- | --- |
| **Hydrophobic Interactions** | | | | | |
| DprE1 | 272 | Leu | DprE2 | 113 | Val |
| DprE1 | 272 | Leu | DprE2 | 115 | Ile |
| DprE1 | 278 | Val | DprE2 | 113 | Val |
| DprE1 | 279 | Phe | DprE2 | 113 | Val |
| DprE1 | 279 | Phe | DprE2 | 159 | Val |
| DprE1 | 280 | Pro | DprE2 | 98 | Leu |
| DprE1 | 287 | Tyr | DprE2 | 197 | Met |
| DprE1 | 291 | Pro | DprE2 | 201 | Leu |
| DprE1 | 292 | Ile | DprE2 | 201 | Leu |
| **Ionic interactions** | | | | | |
| DprE1 | 58 | Arg | DprE2 | 104 | Glu |
| DprE1 | 277 | Asp | DprE2 | 155 | Arg |
| DprE1 | 286 | Lys | DprE2 | 101 | Asp |
| DprE1 | 286 | Lys | DprE2 | 203 | Glu |
| DprE1 | 325 | Arg | DprE2 | 104 | Glu |
| **Hydrogen bonding** | | | | | |
| DprE1 | 58 | Arg | DprE2 | 104 | Glu |
| DprE1 | 277 | Asp | DprE2 | 101 | Asp |
| DprE1 | 277 | Asp | DprE2 | 102 | Ala |
| DprE1 | 277 | Asp | DprE2 | 155 | Arg |
| DprE1 | 277 | Asp | DprE2 | 157 | Asn |
| DprE1 | 286 | Lys | DprE2 | 101 | Asp |
| DprE1 | 286 | Lys | DprE2 | 203 | Glu |
| DprE1 | 322 | Glu | DprE2 | 103 | Glu |
| DprE1 | 322 | Glu | DprE2 | 105 | Leu |

**d.**

| **Molecule** | **Residue number** | **Residue code** | **Molecule** | **Residue number** | **Residue code** |
| --- | --- | --- | --- | --- | --- |
| **Hydrophobic Interactions** | | | | | |
| DprE1 | 275 | Leu | DprE2 | 96 | Phe |
| DprE1 | 276 | Pro | DprE2 | 115 | Ile |
| DprE1 | 276 | Pro | DprE2 | 118 | Ile |
| DprE1 | 276 | Pro | DprE2 | 98 | Leu |
| DprE1 | 278 | Val | DprE2 | 42 | Leu |
| DprE1 | 284 | Ala | DprE2 | 118 | Ile |
| DprE1 | 326 | Ala | DprE2 | 124 | Val |
| DprE1 | 327 | Tyr | DprE2 | 120 | Tyr |
| **Ionic interactions** | | | | | |
| DprE1 | 12 | Arg | DprE2 | 132 | Glu |
| DprE1 | 277 | Asp | DprE2 | 47 | Arg |
| DprE1 | 286 | Lys | DprE2 | 117 | Glu |
| DprE1 | 325 | Arg | DprE2 | 69 | Asp |
| **Hydrogen bonding** | | | | | |
| DprE1 | 18 | Arg | DprE2 | 127 | Gly |
| DprE1 | 58 | Arg | DprE2 | 74 | Asp |
| DprE1 | 277 | Asp | DprE2 | 47 | Arg |
| DprE1 | 281 | Asn | DprE2 | 114 | Gln |
| DprE1 | 284 | Ala | DprE2 | 114 | Gln |
| DprE1 | 286 | Lys | DprE2 | 117 | Glu |
| DprE1 | 327 | Tyr | DprE2 | 120 | Tyr |

**e.**

| **Molecule** | **Residue number** | **Residue code** | **Molecule** | **Residue number** | **Residue code** |
| --- | --- | --- | --- | --- | --- |
| **Hydrophobic Interactions** | | | | | |
| DprE1 | 273 | Leu | DprE2 | 106 | Trp |
| DprE1 | 276 | Pro | DprE2 | 105 | Leu |
| DprE1 | 276 | Pro | DprE2 | 206 | Leu |
| DprE1 | 280 | Pro | DprE2 | 105 | Leu |
| DprE1 | 280 | Pro | DprE2 | 206 | Leu |
| DprE1 | 283 | Leu | DprE2 | 113 | Val |
| DprE1 | 283 | Leu | DprE2 | 115 | Ile |
| DprE1 | 283 | Leu | DprE2 | 116 | Ala |
| DprE1 | 283 | Leu | DprE2 | 158 | Phe |
| DprE1 | 287 | Tyr | DprE2 | 98 | Leu |
| DprE1 | 287 | Tyr | DprE2 | 115 | Ile |
| DprE1 | 287 | Tyr | DprE2 | 118 | Ile |
| DprE1 | 323 | Trp | DprE2 | 102 | Ala |
| DprE1 | 327 | Tyr | DprE2 | 197 | Met |
| DprE1 | 327 | Tyr | DprE2 | 201 | Leu |
| **Ionic interactions** | | | | | |
| DprE1 | 18 | Arg | DprE2 | 203 | Glu |
| DprE1 | 286 | Lys | DprE2 | 101 | Asp |
| DprE1 | 286 | Lys | DprE2 | 104 | Glu |
| **Hydrogen bonding** | | | | | |
| DprE1 | 18 | Arg | DprE2 | 203 | Glu |
| DprE1 | 272 | Leu | DprE2 | 107 | Gln |
| DprE1 | 272 | Leu | DprE2 | 108 | Asn |
| DprE1 | 274 | Thr | DprE2 | 105 | Leu |
| DprE1 | 274 | Thr | DprE2 | 106 | Trp |
| DprE1 | 280 | Pro | DprE2 | 155 | Arg |
| DprE1 | 281 | Asn | DprE2 | 104 | Glu |
| DprE1 | 281 | Asn | DprE2 | 106 | Trp |
| DprE1 | 281 | Asn | DprE2 | 157 | Asn |
| DprE1 | 282 | Gly | DprE2 | 106 | Trp |
| DprE1 | 286 | Lys | DprE2 | 102 | Ala |
| DprE1 | 327 | Tyr | DprE2 | 196 | Arg |

**f.**

| **Molecule** | **Residue number** | **Residue code** | **Molecule** | **Residue number** | **Residue code** |
| --- | --- | --- | --- | --- | --- |
| **Hydrophobic Interactions** | | | | | |
| DprE1 | 267 | Phe | DprE2 | 106 | Trp |
| DprE1 | 27prE22 | Leu | DprE2 | 106 | Trp |
| DprE1 | 275 | Leu | DprE2 | 113 | Val |
| DprE1 | 275 | Leu | DprE2 | 159 | Val |
| DprE1 | 276 | Pro | DprE2 | 98 | Leu |
| DprE1 | 276 | Pro | DprE2 | 159 | Val |
| DprE1 | 278 | Val | DprE2 | 113 | Val |
| DprE1 | 287 | Tyr | DprE2 | 201 | Leu |
| DprE1 | 292 | Ile | DprE2 | 201 | Leu |
| DprE1 | 326 | Ala | DprE2 | 206 | Leu |
| DprE1 | 327 | Tyr | DprE2 | 204 | Ala |
| DprE1 | 327 | Tyr | DprE2 | 206 | Leu |
| DprE1 | 327 | Tyr | DprE2 | 212 | Tyr |
| DprE1 | 330 | Ala | DprE2 | 213 | Val |
| **Ionic interactions** | | | | | |
| DprE1 | 18 | Arg | DprE2 | 211 | Glu |
| DprE1 | 119 | Arg | DprE2 | 104 | Glu |
| DprE1 | 286 | Lys | DprE2 | 101 | Asp |
| DprE1 | 286 | Arg | DprE2 | 203 | Glu |
| **Hydrogen bonding** | | | | | |
| DprE1 | 18 | Arg | DprE2 | 211 | Glu |
| DprE1 | 119 | Arg | DprE2 | 104 | Glu |
| DprE1 | 277 | Asp | DprE2 | 114 | Gln |
| DprE1 | 277 | Asp | DprE2 | 115 | Ile |
| DprE1 | 286 | Lys | DprE2 | 101 | Asp |
| DprE1 | 286 | Lys | DprE2 | 203 | Glu |
| DprE1 | 326 | Ala | DprE2 | 154 | Arg |
| DprE1 | 327 | Tyr | DprE2 | 204 | Ala |
| DprE1 | 327 | Tyr | DprE2 | 211 | Glu |
| DprE1 | 330 | Ala | DprE2 | 214 | Ala |
